# The unique functioning of a pre-Columbian Amazonian floodplain fishery

**DOI:** 10.1038/s41598-018-24454-4

**Published:** 2018-04-16

**Authors:** Rumsaïs Blatrix, Bruno Roux, Philippe Béarez, Gabriela Prestes-Carneiro, Marcelo Amaya, Jose Luis Aramayo, Leonor Rodrigues, Umberto Lombardo, Jose Iriarte, Jonas Gregorio de Souza, Mark Robinson, Cyril Bernard, Marc Pouilly, Mélisse Durécu, Carl F. Huchzermeyer, Mashuta Kalebe, Alex Ovando, Doyle McKey

**Affiliations:** 1 CEFE, CNRS, University of Montpellier, University Paul Valéry Montpellier 3, EPHE, IRD, 1919 route de Mende, 34293 Montpellier, France; 2 L’Avion Jaune, 1 chemin du Fescau, 34980 Montferrier-sur-Lez, France; 3 Archéozoologie, Archéobotanique: Sociétés, Pratiques et Environnements (UMR 7209), Sorbonne Université, CNRS, Muséum National d’Histoire Naturelle, CP 56, 75005 Paris, France; 4 Museo de Historia Natural Noel Kempff Mercado, Department of Entomology, U.A.G.R.M., P. O. Box 702, Santa Cruz de la Sierra, Bolivia; 5 CaSEs – Complexity and Socio-Ecological Dynamics Research Group, Department of Humanities, Pompeu Fabra University. Ramon Trias Fargas 25-27, Mercè Rodoreda Building, 08005 Barcelona, Spain; 6 Department of Archaeology, University of Exeter, Laver Building, North Park Road, Exeter, EX4 4 QE United Kingdom; 7 Institut de Recherche pour le Développement, UMR BOREA (Muséum National d’Histoire Naturelle, l’Institut de Recherche pour le Développement, UMR 7208, CNRS, Université Pierre et Marie Curie, Université de Caen Normandie), 75231 Paris, France; 8 Bangweulu Wetlands Project, African Parks Network, Mpika, Zambia; 9 Mt. Makulu Central Research Station, Zambia Agricultural Research Institute, Chilanga, Zambia; 10 National Centre for Monitoring and Early Warning of Natural Disasters (CEMADEN), Estrada Doutor Altino Bondesan, 500 - Distrito de Eugênio de Melo, São José dos Campos/SP, Brazil, Sao Paulo, Brazil

## Abstract

Archaeology provides few examples of large-scale fisheries at the frontier between catching and farming of fish. We analysed the spatial organization of earthen embankments to infer the functioning of a landscape-level pre-Columbian Amazonian fishery that was based on capture of out-migrating fish after reproduction in seasonal floodplains. Long earthen weirs cross floodplains. We showed that weirs bear successive V-shaped features (termed ‘Vs’ for the sake of brevity) pointing downstream for outflowing water and that ponds are associated with Vs, the V often forming the pond’s downstream wall. How Vs channelled fish into ponds cannot be explained simply by hydraulics, because Vs surprisingly lack fishways, where, in other weirs, traps capture fish borne by current flowing through these gaps. We suggest that when water was still high enough to flow over the weir, out-migrating bottom-hugging fish followed current downstream into Vs. Finding deeper, slower-moving water, they remained. Receding water further concentrated fish in ponds. The pond served as the trap, and this function shaped pond design. Weir-fishing and pond-fishing are both practiced in African floodplains today. In combining the two, this pre-Columbian system appears unique in the world.

## Introduction

Although archaeological artefacts document diverse fish-capturing gear, from spears and hooks to nets, traps and weirs^1,2^, and rock art depicts fishing scenes, including fish traps^3–5^, the large-scale ecological functioning of fisheries and its consequences for the social organisation of fishing activities in the past are often difficult to infer. Some studies have used large collections of fish remains such as bones, scales and otoliths^6^ to document the species harvested and attempt to infer how fishing activities may have been organised^7,8^, using knowledge about the biology and ecology of the harvested fish species. However, for many parts of the world such studies are rare. In Amazonia, for example, despite the great productive potential of river and floodplain fisheries and the contemporary importance of inland fisheries in the region^9^, there has been little investigation of fisheries in the past.

Only rarely do archaeological remains permit functional analysis of large-scale sophisticated fish-harvesting systems^10^. The study by Greene *et al*.^11^ of a weir-based estuarine fishery on Canada’s Pacific coast is such an example. This fishery relied on tidal fluctuations and the fish movements associated with them. During the ebbing tide, fish encountered fences made of wooden poles that led to enclosures. Greene *et al*.’s analysis of human modifications of the landscape, combined with knowledge of fish behaviour and ecology, enabled them to make inferences about the system’s functioning. Clam gardens provide another example where archaeological vestiges, combined with experimental studies of present-day reconstructions, yield insights into the functioning of a sophisticated coastal fishery^12–15^.

Inland fisheries have long played an important role in subsistence of hunter-gatherer and agricultural societies, as documented by abundant fish remains in many archaeological settings^2,6,16,17^. However, few studies help us understand the ecological functioning of these fisheries, particularly those systems at the frontier between catching of fish and farming of fish that some have termed ‘proto-aquaculture’^10^. The best-studied example appears to be human-modified wetlands around Lake Condah in Australia, where networks of channels (up to 3.5 km long) dug through sediments and lava flows are linked to elaborate trapping facilities and ponds^18–22^ that appear to have been focused on a single fish species, the short-finned eel, *Anguilla australis*^23^.

A second archaeological example of a large-scale, relatively intensive inland fishery is the pre-Columbian floodplain fishery based on an extensive network of earthen fish weirs and ponds that was described by Erickson^24–26^ within a localised region of Bolivia’s Llanos de Moxos in southwestern Amazonia. (In keeping with common practice, ‘pre-Columbian’ is used here to refer to indigenous cultures in the Americas before their extensive alteration by contact with Europeans, which in some regions occurred long after 1492). The fishery was abandoned at least 300 years ago^24^. The closest ecological and cultural analogue of this system appears to be a present-day floodplain fishery, also based on earthen weirs, in Zambia’s Bangweulu Basin in southern Africa. McKey *et al*.^27^ demonstrated general convergence in how societies in these environments constructed the fishery dimension of their ecological niche. Although geographically widely separated, the two environments are ecologically similar in rainfall and flooding regime and the fish communities of both environments include periodically breeding fish that migrate laterally between the floodplain and the river. In the pre-Columbian South American system, these species, like their African analogues today^27^, could have been sustainably exploited by weirs during their annual out-migration from floodplains. In both systems, weirs function to channel fish movement, with V-shaped structures concentrating fish at particular points where they can be much more easily captured than if they were dispersed throughout the vast floodplain.

Archaeological inferences about past subsistence systems cannot provide the detailed information that can be obtained by observation of present-day systems. We thus know little about how the archaeological fishery functioned, compared to what we can learn from direct observation of the present-day fishery^27^. Nevertheless, precise description of the material vestiges of past subsistence systems can help guide inferences about how they functioned, and thus show how they resembled, and differed from, present-day systems.

Ecological niche construction by humans has shaped biodiversity and ecosystem patterns at the global scale for more than 10,000 years^28^. The making of weirs to channel fish movements is a frequent component of niche construction^29^. To what extent was the fishery of the Llanos de Mojos shaped by environmental factors such as flood regime? To what extent did niche-construction activities within this fishery affect local distributions of fish and plant species? Most fundamentally, how did these earthworks function to trap fish? From existing descriptions, this central point is still unclear. Knowing in detail how this fishery functioned is key to understanding how humans adapted to the floodplain environment and how they impacted it. To produce a more precise description of the floodplain landscape, and to infer from this description how the fishery functioned, we conducted fieldwork in the San Joaquín floodplains, within the Blanco-San Martín basin, where these human-made earthen structures were first described by Erickson^24^. Ground observation of topography for a number of earthen structures allowed interpretation of topography for many more structures from satellite images and from aerial photographs taken during flyovers in a small plane. Finally, we illustrated the topography of ponds, pond rims and V-shaped structures of weirs by stereophotogrammetry, using series of photographs taken with a kite-borne camera. Our spatially explicit quantitative analysis of the landscape suggests a compelling hypothesis about how this fishery functioned.

## Results

### The nature of vestiges of anthropogenic features

**Figure 1:**
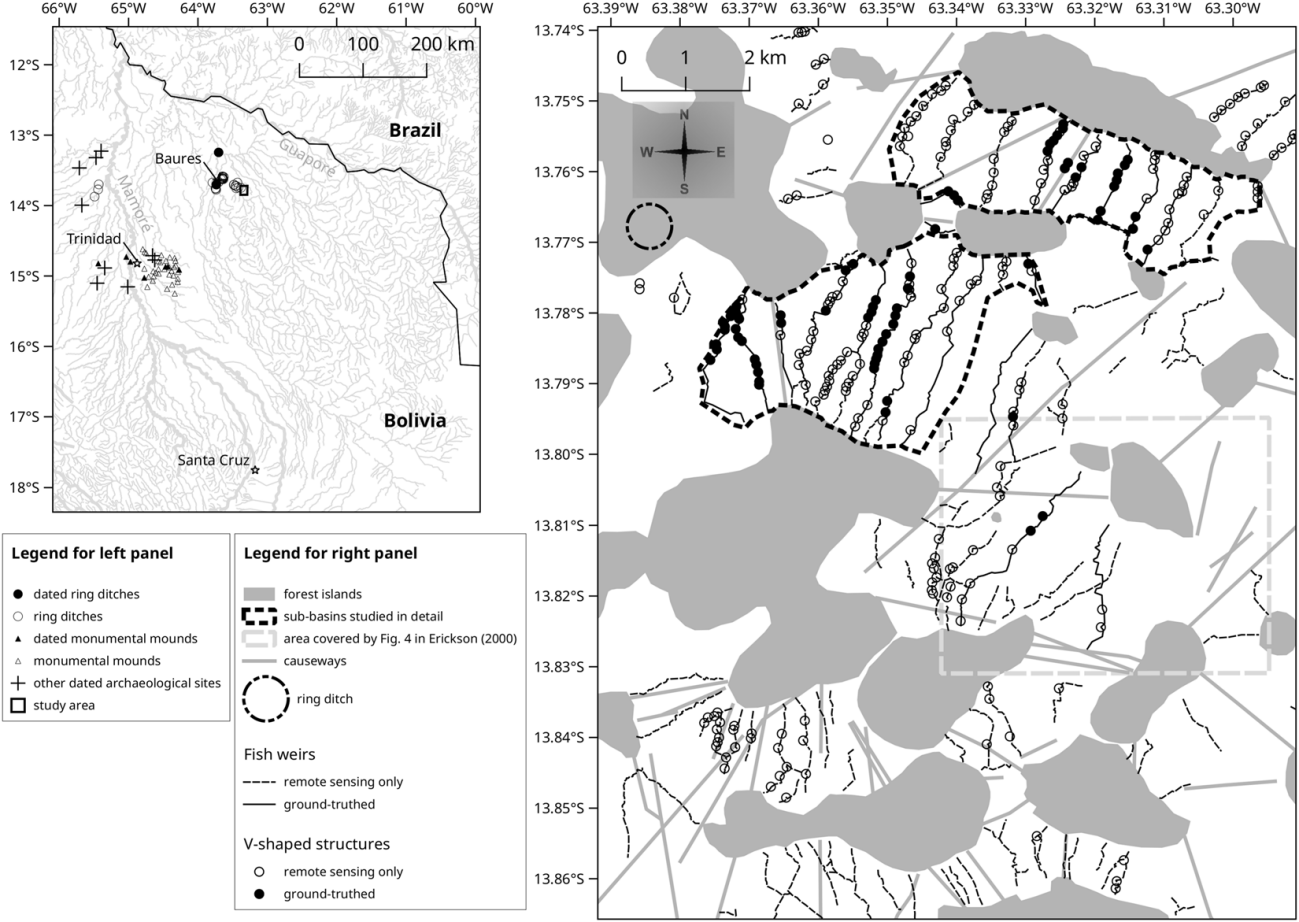
Distribution of weirs and V-shaped structures in the studied area. Maps were generated with QGIS 2.18 (www.qgis.org).

**Figure 2:**
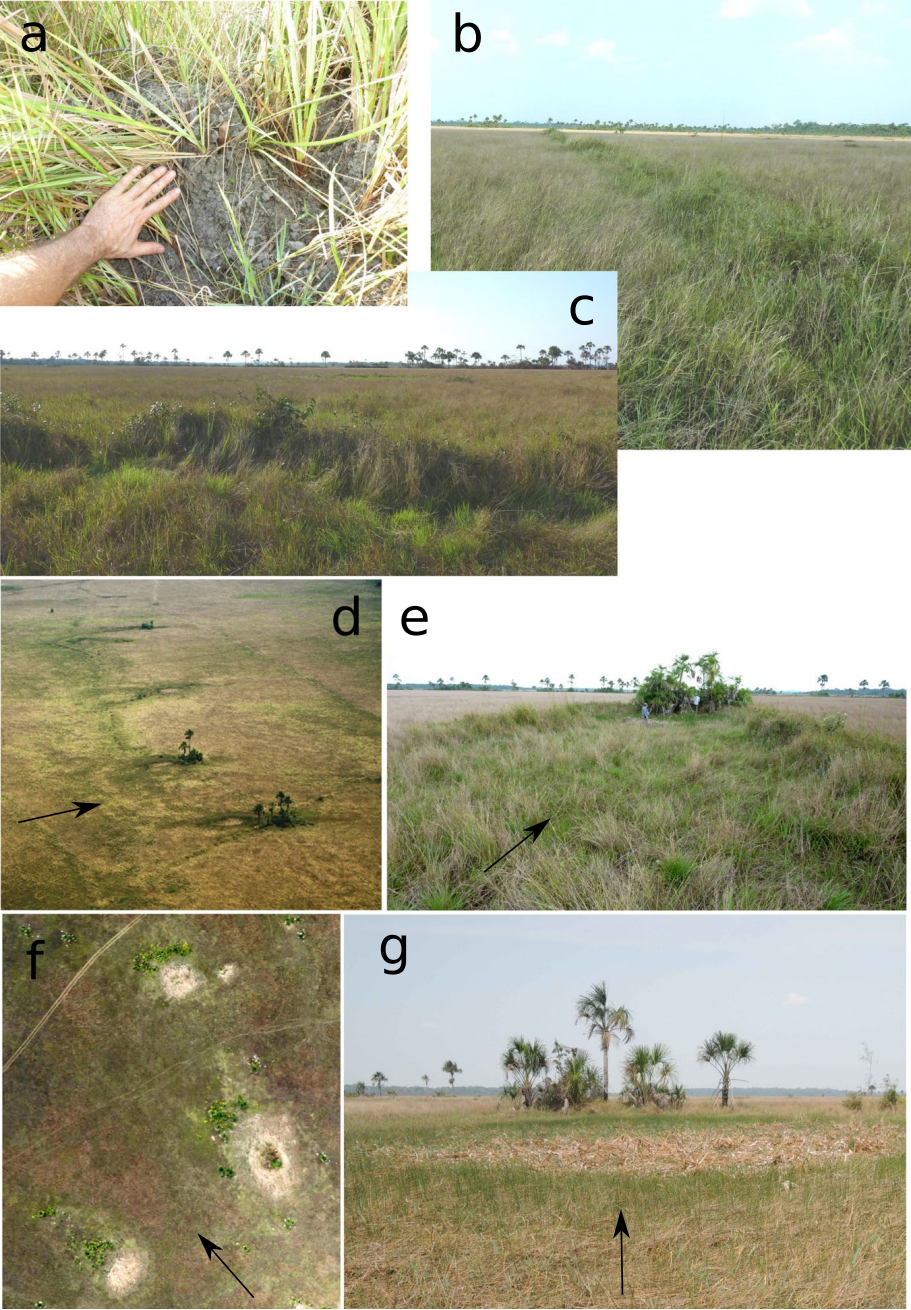
Vestiges of pre-Columbian earthworks are still visible today. (**a**) Accumulations of earthworm casts cover the surfaces of weirs. Vestiges of weirs have been extensively reworked by earthworms, which are attracted to these and other elevated structures during the flooding period. (**b**,**c**) Weirs are still visible not only because earthworms continually move soil to them, but also because they harbour taller vegetation (tall grass and woody plants) than the lower-lying floodplain. (**d**) Aerial photo of four successive V-shaped structures along a weir, showing associated (dry) ponds, and woody vegetation at the point of each V. (**e**) V-shaped structure seen from the ground, looking downstream, showing trees and dense vegetation growing at the elevated point of the V, and a pond just upstream of the point. (**f**) Aerial orthophoto of three (dry) ponds, the downstream berm of each of which is colonised by woody vegetation. (**g**) Ground view of a dry pond. Arrows indicate direction of water flow during the high-water season. Photos (11–17 October 2016): Rumsaïs Blatrix (**a**–**c**,**e**), Bruno Roux (**d**,**f**) and Doyle McKey (**g**).

The San Joaquín floodplains are criss-crossed by vestiges of zigzag weirs and of arrow-straight causeways (Fig. 1, Supplementary Fig. S1) that allowed people to walk from one forest island to another^24^. The zigzag pattern of weirs often results from successive V-shaped indentations of the embankment (Fig. 2). Erickson^24–26^ suggested the existence of a gap at the point of each of these V-shaped structures, that would have been of appropriate size for the placement of a basket or trap to catch fish. The floodplains are dotted with numerous ponds considered by Erickson^24–26^ to have been made by humans and related to weirs. The principal study area of Erickson^24^ (see Fig. 1) is overgrown with trees and shrubs, which nowadays occur not only on the earthworks but also on neighbouring lower-lying areas. Earthworks, including earthen weirs, are thus difficult to observe and interpret. We found that in areas located slightly further north, woody vegetation was much less well developed in the floodplain and observations of earthworks were clearer. We delimited in this area the two sub-basins that we studied in detail (Fig. 1). We call these two study areas “sub-basins” because each represents a continuous grassland bound on two sides by natural elevations (outcrops of the Brazilian shield that are covered by forest and thus termed ‘forest islands’) and in which water in the high-water season flows in the same direction throughout the sub-basin (Supplementary Fig. S2).

Soil organisms interact with the vestiges of human earth-moving activity, sometimes obscuring them^30,31^, sometimes playing an essential role in their preservation^32^. Vestiges of weirs and causeways persist because soil organisms (a) establish preferentially on these elevated structures and (b) combat the erosion of these structures by accumulation of materials and production of stabilizing biostructures (Fig. 2a). Consequently, although vestiges of weirs are only 10–40 cm higher than floodplain ground, they are distinguished by the taller grassy vegetation and by the scattered presence of woody plants, which in our study site were absent from lower ground (Fig. 2b–e, Supplementary Fig. S3). Pond berms and causeways, both of which are higher than weirs, harboured large numbers of palm trees (Fig. 2f,g, Supplementary Fig. S1). Vegetation distribution may have differed somewhat in pre-Columbian times. Nevertheless, current distribution of woody plants, after 300 years without human management, is a good proxy for higher ground^33^.

### Ponds are spatially associated with weirs and causeways

**Figure 3:**
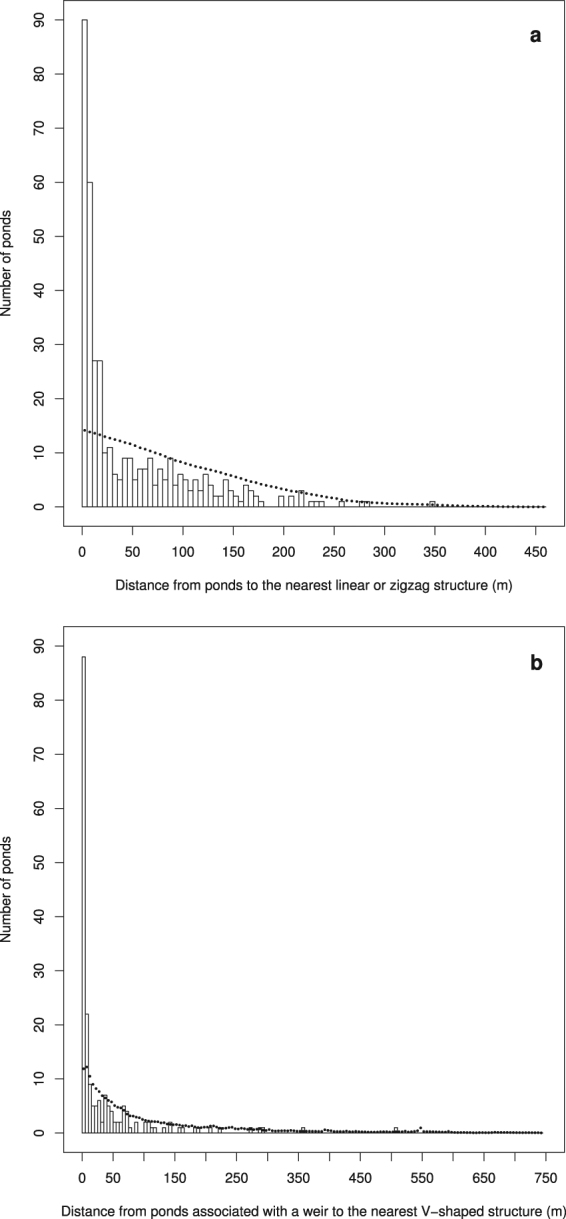
Distribution of the distances between ponds and the nearest linear anthropogenic structure (causeway, canal or weir) (**a**), and between ponds associated with weirs and the nearest V-shaped structure (**b**). White bars represent the distribution of observed distances. Black dots represent the mean values for 10,000 simulated random distributions of the same number of ponds (vertical bars representing standard deviations do not exceed the radius of the dots and are not visible).

The distribution of the distances from pond centroids to the nearest linear (causeways) or zigzag (weirs) anthropogenic structure was skewed toward shorter distances (Fig. 3a), revealing that most ponds are spatially associated with these features: 231 (60%) of the 382 ponds that we detected in the sub-basins studied are within 35 m of such a feature, most of which (191 of 231, i.e. 83%) are within 35 m of a weir (the others being within 35 m of a causeway). The probability that a random distribution of the ponds would give more ponds within 35 m of a linear or zigzag structure than observed is <0.0001, based on 10,000 simulations of 382 ponds randomly distributed in the studied sub-basins. For the association of ponds with weirs to become non-significant (probability >0.05), over 470 ponds more than 35 m from a linear or zigzag linear anthropogenic structure would have had to escape notice during our inspection of aerial images and our fieldwork (see Supplementary Fig. S4). We are thus confident that ponds are significantly associated with linear and zigzag anthropogenic features.

The distribution of the distances from ponds to the nearest V-shaped structure of a weir is also skewed toward shorter distances (Fig. 3b). Considering only those ponds spatially associated with a weir (zigzag feature), centroids of 137 out of 191 (i.e. 72%) are within 35 m of a V-shaped structure and thus are also spatially associated with a V-shaped structure in the weir. The probability that a random distribution of the ponds would give more ponds within 35 m of a V-shaped structure than observed is <0.0001, based on 10,000 simulations of 191 ponds randomly distributed along weirs in the studied sub-basins.

### V-shaped structures point downstream relative to the direction of receding water, have ponds associated mostly upstream and are elevated at the point of the V

**Figure 4:**
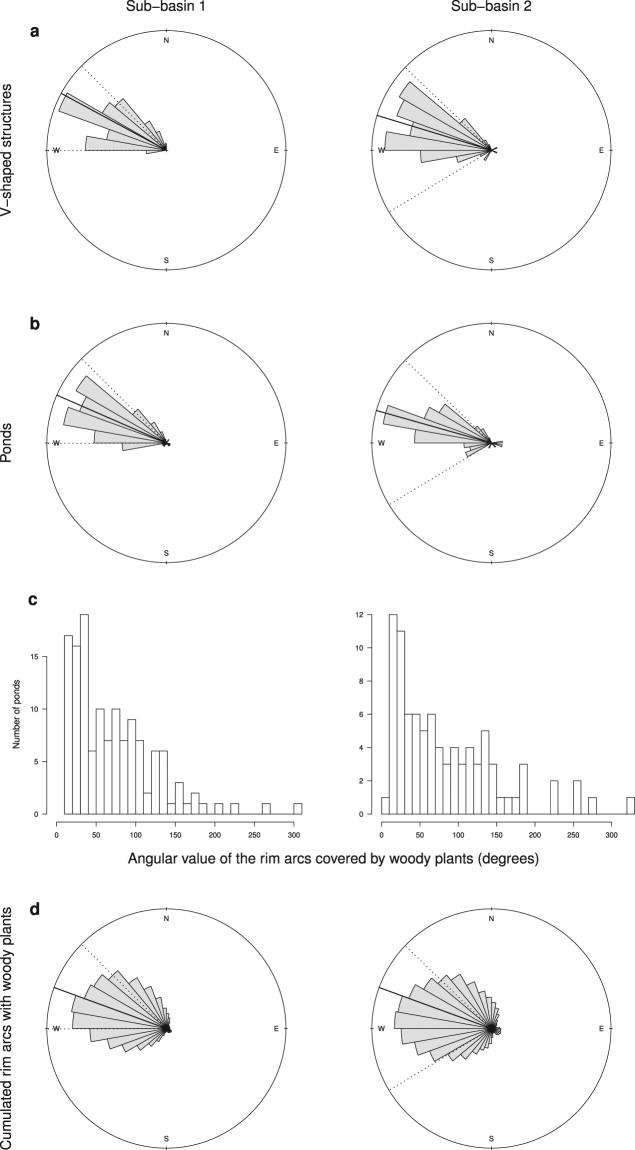
Distribution of the orientation of V-shaped structures (from the widest part to the point of the V) (**a**) and of ponds (both those associated with linear structures and those that are isolated) from the lowest point in the centre to the highest point of the berm (**b**), in the two sub-basins. (**c**) Size distribution of the angle of the arc formed by woody plant presence around ponds. (**d**) Distribution of the orientation of rim arcs covered with woody plants, cumulated over all ponds. Solid lines represent the mean azimuth for each distribution. Dotted lines represent the extreme values of the putative direction of water flow during the high-water season for each sub-basin.

**Figure 5:**
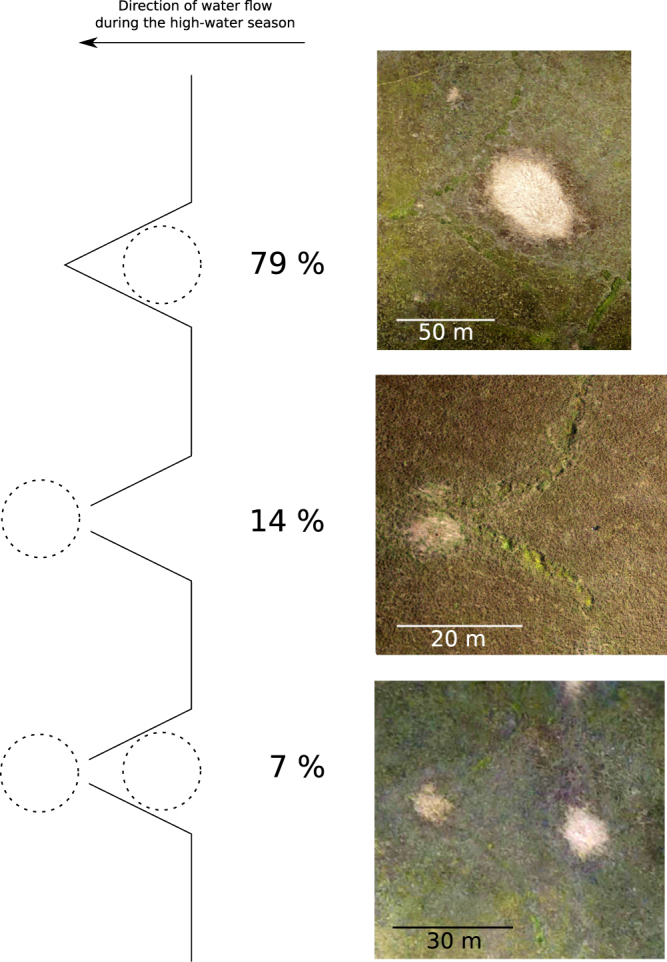
Schematic representation of the three main types of position of ponds relative to the V-shaped structures.

V-shaped structures in the sub-basins are not oriented randomly (Kuiper test; sub-basin 1: statistic = 6.55, P < 10^−36^, n = 71; sub-basin 2: statistic = 8.28, P < 10^−58^, n = 101). They are all oriented in the same direction, following the direction of water flow during the high-water season, the point of the V pointing downstream (Fig. 4a). Out of the 270 V-shaped structures we detected in total (98 are outside the sub-basins), 244 (i.e. 90%) had no gap at the point of the V, as they would if they had functioned as fishways. For the others, a gap appeared to be present at the point. However, these apparent gaps were usually much too wide to have accommodated a basket or trap. Some may have functioned to channel water into a pond (see Fig. 5b); others may simply have resulted from erosion of the weir.

For 133 (i.e. 49%) V-shaped structures we could not detect any associated pond (see Supplementary Fig. S5). We probably underestimated the number of V-shaped structures that were associated with ponds because (as noted above) ponds become difficult to detect from aerial imagery as they are filled in with sediment over time. Out of the 137 V-shaped structures for which we detected an associated pond, 109 (i.e. 79%) had one or two associated ponds upstream, nine (i.e. 7%) had one pond upstream and one pond downstream, and 19 (i.e. 14%) had one pond downstream (Fig. 5).

**Figure 6:**
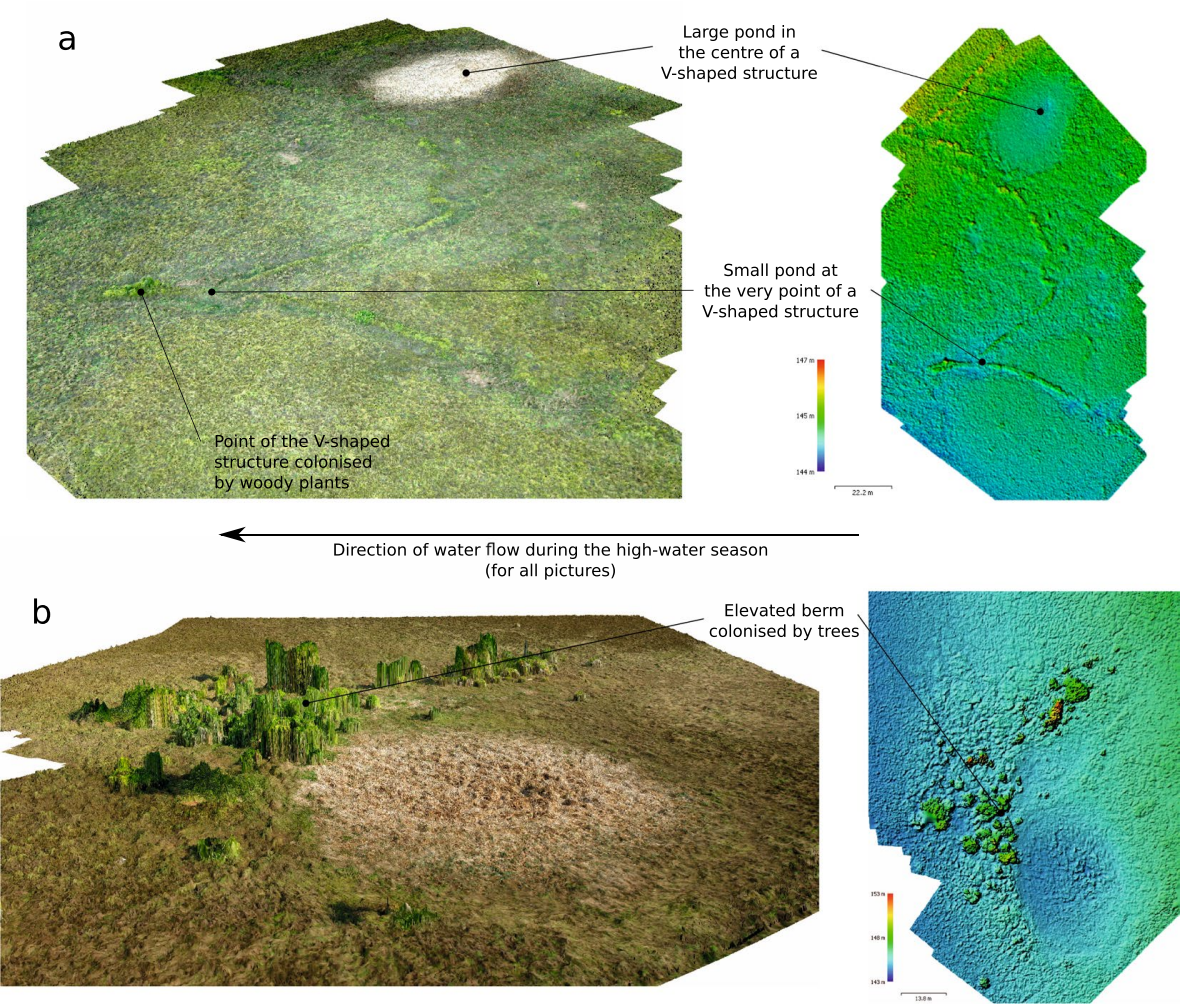
Digital surface models and oblique views of textural 3D models reconstructed by stereophotogrammetry, using series of photographs taken with a kite-borne camera. (**a**) Two V-shaped structures along a weir, one (in the foreground) with a small pond at the very point of the V, the other with a large pond in the centre of the V, both with elevated ground at the point. (**b**) A pond with a downstream berm colonised by trees.

In most cases there is no gap at the point of the V. The mean height difference (±s.d.), measured manually in the field, between the highest soil level at the point of the V and the lowest part of the surface of the enclosed pond is 0.79 ± 0.25 m (n = 13). In fact, the point of the V is the *highest* part of the weir, often harbouring palms and other woody vegetation, whereas the rest of the weir is somewhat lower and harbours mostly grasses (Figs 2d,e, 6a, Supplementary Video 1).

### Berms of ponds are highly asymmetrical, concentrated on the downstream side

Of the 382 ponds mapped in the sub-basins, we made direct observations on the ground of 89 ponds. Sixty-seven of these had berms, elevated areas around the rim that are usually colonized by palms and other woody vegetation (evident on aerial images). For ponds not associated with V-shaped structures (for the others see previous section), the mean height difference (±s.d.), measured manually in the field, between the highest soil level of the berm and the centre of the pond was 1.18 ± 0.22 m (n = 6). For 293 other ponds we had only remotely sensed data (aerial photographs and satellite imagery). For 139 of these others, we could not determine from the images whether or not a berm was present. For the 221 ponds where a berm was evident from field observations, remote sensing or both, the distribution of the direction of the highest point of the berm was not random (Kuiper test; sub-basin 1: statistic = 7.38, P < 10^−46^, n = 134; sub-basin 2: statistic = 6.40, P < 10^−34^, n = 87). The highest points of berms are all oriented in the same direction, following the direction of water flow during the high-water season (Figs 2f, 4b). The orientation of pond berms is not different from that of the V-shaped structures (Watson-Williams test; sub-basin 1: P = 0.21; sub-basin 2: P = 0.81).

As presented above, ponds associated with V-shaped structures are usually just *upstream* of these structures. In fact, the point of the V is usually part of the highest part of the berm of earth associated with the pond (Fig. 2d,e, Supplementary Video 1). Palms and other woody plants do not surround each pond, as described by Erickson & Brinkmeier^26^, but rather occupy only a small portion of the pond rim, as shown by the values of the angle of the rim covered by woody plants (Figs 2f,g, 4c, 6b). The distribution is skewed toward small values. Moreover, the arcs of woody plants, and thus of the berms around ponds, are not distributed randomly (Kuiper test; sub-basin 1: statistic = 49, P = 0; sub-basin 2: statistic = 34, P = 0). This means that ponds have short berms all oriented in the same direction, i.e. on the *downstream* side of the pond (Fig. 4d), where the berm of soil is highest, suggesting that the orientation of berms is related to the direction of water flow during the high-water season. This characteristic orientation of berms holds both for ponds isolated in savannah and for ponds associated with weirs and causeways (Fig. 6, Supplementary Fig. S6). As stated above, in ponds associated with V-shaped structures of weirs, the berm often merges with the point of the V, which is the highest part of the V.

### V-shaped structures are larger and relatively wider in pre-Columbian Bolivian weirs than in present-day Zambian weirs

**Figure 7:**
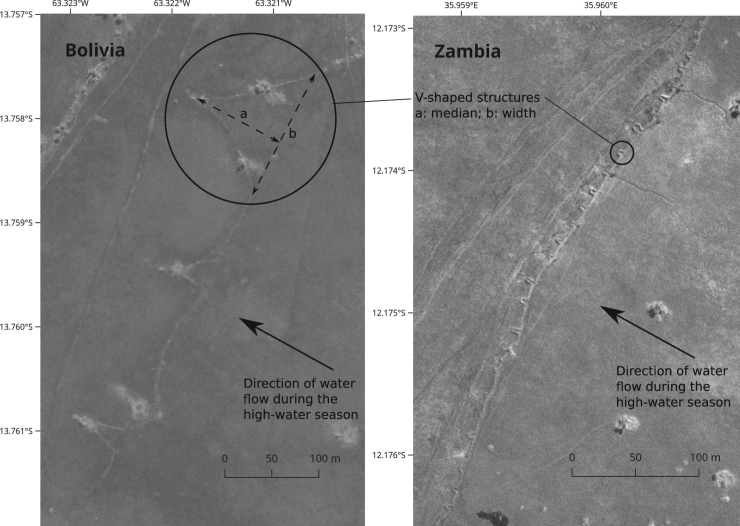
Comparison of the V-shaped structures on weirs in Bolivia (World Imagery ArcGIS layer, ESRI, Digital Globe, June 2008) and Zambia (satellite image by the Pléiades sensor, July 2013. Pléiades © CNES 2013, distribution Airbus DS). This figure is not covered by the CC BY licence. All rights reserved, used with permission.

V-shaped structures of weirs are much smaller in the Bangweulu Basin in Zambia (length of the median = 5.3 ± 2.4 m, width [maximum distance between the arms] = 3.7 ± 1.5 m, means ± s.d., n = 100) than in the San Joaquín floodplain in Bolivia (length = 30.0 ± 18.5 m, width = 38.5 ± 34.3 m, means ± s.d., n = 100) (Fig. 7, with illustration of measurements; *t*-test; length: *t* = 13, p < 10^−23^; width: *t* = 10, P < 10^−16^). The shape of Vs is also different between the two systems: the length/width ratio is larger in Zambia (means ± s.d. = 1.60 ± 0.98) than in Bolivia (means ± s.d. = 0.98 ± 0.49) (*t*-test; *t* = 6, P < 10^−7^) (Fig. 7). Furthermore, whereas the pre-Columbian Bolivian weirs lack fishway gaps, a gap (mean width ± s.d. = 1.0 ± 0.3 m) is always present at the point of each V in the Zambian weirs.

### Fish-concentration potential is lower in pre-Columbian Bolivian weirs than in present-day Zambian weirs

In the San Joaquín floodplain in Bolivia, ponds have a mean (±s.d.) surface area of 525 ± 548 m^2^ (n = 382). For comparison, traps fitted to fishways in the Bangweulu Basin in Zambia have entrance hoops 0.3 to 1.5 m in diameter^34^. In Bolivia, the ratio between the surface of capture area between successive weirs and the surface of collecting structures (ponds) in the capture area varied between 27 to 193, with a mean ± s.d. of 87 ± 42 (n = 15 inter-weir areas). This is several orders of magnitude lower than the concentration potential of the fishery in Zambia, where the ratio between the surface of the capture area and the total surface of interception of the traps receiving water from the capture area varied between 9,741 to 78,588, with a mean ± s.d. of 36,154 ± 18,353 (n = 15 inter-weir areas). The surface of capture areas between successive weirs was approximately twice as large in Bolivia (383,702 to 1,778,073 m^2^) as in Zambia (53,554 to 842,075 m^2^).

### Observations from a transect excavated in a pond

**Figure 8:**
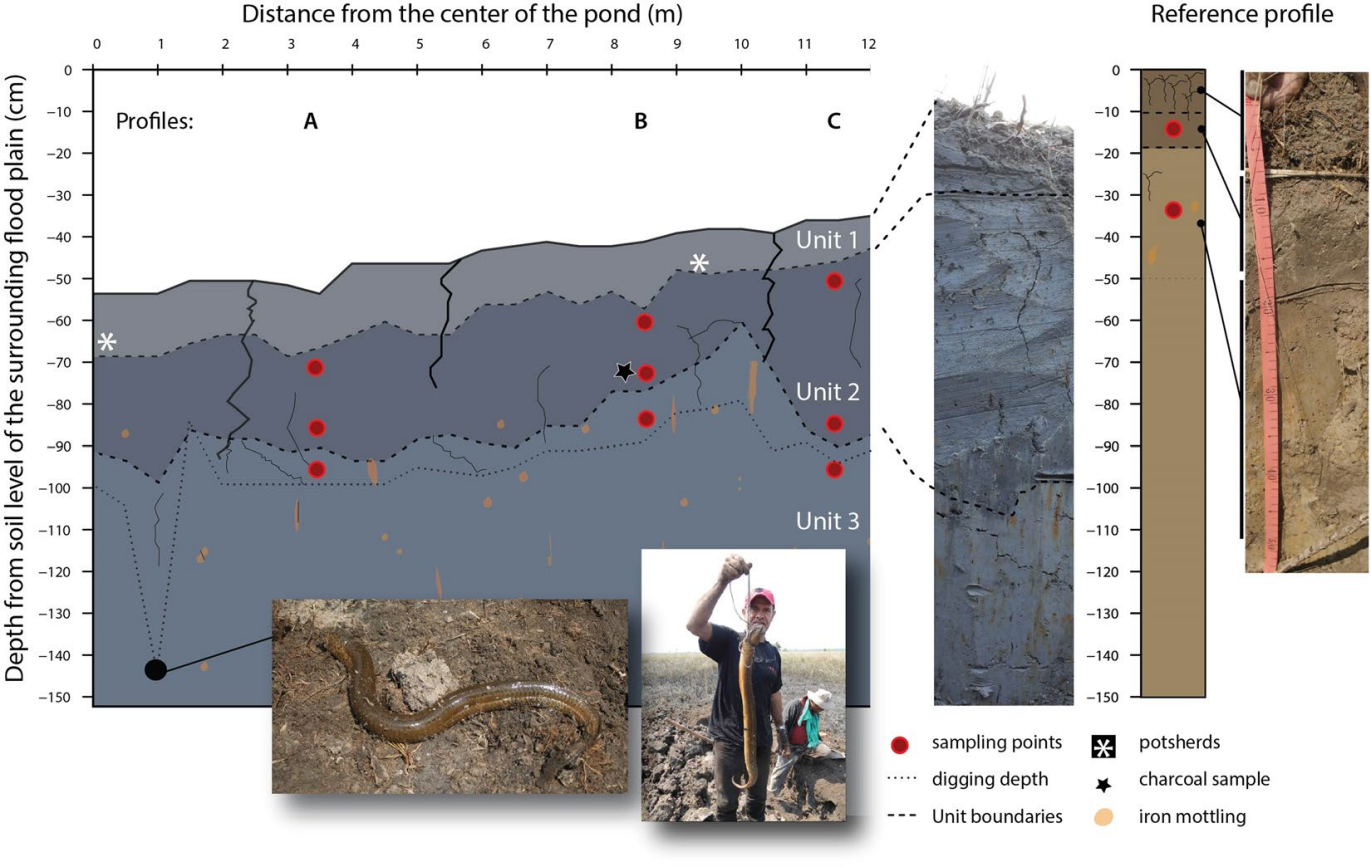
Soil profile for a 14-m-long trench in a pond. The depth 0 m represents the ground level in the floodplain outside the pond. Solid line: ground level; dashed lines: soil units, dotted line: maximum depth dug. Photos (18 October 2016): Bruno Roux and Doyle McKey.

A 14-m-long trench dug in a pond revealed the soil profile (Fig. 8, Supplementary Fig. S7). Three different stratigraphic units with clear boundaries, indicated by sharp colour changes, were identified: Unit 1, the surface soil layer (*c*. 10 cm thick), which consists of loose dark soil rich in roots; Unit 2 (*c*. 28 cm thick), a dense dark layer rich in clay and organic matter; and Unit 3, the bottom layer composed of dense light grey clay with orange mottling, typical of soils with hydromorphic processes. The level of these sediment layers rises gradually from the centre of the pond to the periphery. The modern deepest point of the pond is 55 cm lower than the surrounding plain. Units 1 and 2 show evidence of considerable disturbance, both natural and anthropogenic. Several deep desiccation cracks extend to 30 cm depth, due to repeated wetting and drying of the clays. Such cracks could explain the presence of isolated sand lenses observed in the clay matrix, washed in from the surface to depths of up to 30 cm in the profile. These Units are also highly bioturbated, by plant roots (and probably by large animals such as the fish *Synbranchus* sp., a specimen of which we found during excavation [see the following paragraph]). At the bottom of Unit 2 we found several small pieces (1–5 mm) of charcoal. Radiocarbon analysis of a charcoal fragment and of the humin fraction of a soil bulk sediment sample at the bottom of the in-fill sediment of the pond provided dates between Cal. AD 1030–1180 and Cal. AD 1310–1424, respectively (Supplementary Table S1). The sediments of the reference profile outside the pond are similarly dense and clay-rich. However, their colour is brownish-yellow, indicating lower content of organic matter. The difference in colour between sediments inside and outside the pond is explained by the prolonged anoxic conditions in the pond, hampering the decomposition of the abundant organic matter from the pond’s lusher vegetation and favouring the reduction of oxides of iron and manganese.

While digging we encountered a burrow, *c*. 6 cm in diameter, that we followed. It led us to a swamp eel (*Synbranchus* sp.) at a depth of *c*. 1 m below the surface of the ground in the pond (Fig. 8). At this depth the soil was waterlogged.

While digging the trench in the pond we found two places with pottery sherds (Fig. 8, Supplementary Fig. S7) at a depth of 15–20 cm. We found additional pottery sherds at the same depth in a 2.5 × 2.5 m quadrat excavated in the same pond down to a depth of 30 cm (Supplementary Fig. S7).

## Discussion

Our analysis of spatial patterns, combining remote sensing and ground-truthing, showed that ponds are localised close to weirs and causeways, and that most of them are within V-shaped structures, upstream of the weir. Moreover, V-shaped structures point downstream with the receding water, allowing them to channel the movement of out-migrating fish at the end of the flooding period. Ponds have a berm on the downstream side, merging with the point of the V for those ponds within V-shaped structures. As a result, most V-shaped structures do not have a gap at the point of the V, as they would if they had functioned as fishways. Rather, as flood water subsided, the ponds appear to have served as ‘traps’, where fish could then be predictably contained and harvested. Structural features of ponds appear to be adapted to their trapping function: berms are absent from the upstream rim of ponds, making it easier for out-migrating fish to swim into them, and berms are highest on the downstream rim of ponds, making it more difficult for these fish to swim out of them. Given these functional advantages, one plausible explanation for the observed orientation of berms and woody vegetation is that earth was excavated from a pond and deposited only on its downstream side, and that woody vegetation grew on this higher ground. However, a plausible alternative hypothesis suggesting a natural origin of ponds can also explain the regular orientation of berms with respect to direction of water flow: scouring by water flow around an obstacle, with downstream accretion^35^. This hypothesis is developed in the supplementary information. While it is still too early to draw a conclusion about Erickson’s contention^24^ that these ponds were made by humans, the strong spatial association of ponds with V-shaped features of weirs, along with the topography of ponds and associated berms, provide the first empirical support for his assertion that weirs and ponds functioned as an integrated fish-capturing and fish-storage system. In summary, we postulate that the principal function of the V-shaped structures was to concentrate fish in ponds. For a detailed interpretation of how this integrated system could have worked, see the supplementary information. Sediments from the pond basin and soils from the reference site (seasonal floodplain near the pond) both gave low values for concentration of nutrient elements, particularly phosphorus, which was present in very low concentrations (Supplementary Table S2). The low nutrient status of these soils, and the fact that the floodwaters that cover the San Joaquín floodplains are mostly derived from rainfall (with little fluvial input), should lead to low productivity, enhancing the value of structures that concentrate fish in small areas.

The hypothesis that the pond serves as the trap and that weirs and V-shaped structures guide fish to it can account for the differences in morphology of earthworks between the pre-Columbian fishery in Bolivia and the present-day fishery in Zambia. Although the two landscapes share many similarities, our analysis of the embankments in Bolivia shows that they also differ in important ways from the structures in Zambia. First, in the Zambian landscape there are no ponds, apart from a few scattered megafauna wallows^27^. Second, in the linear or curvilinear weirs of Zambia, V-shaped fishways, where fish are trapped when water flows out of the floodplains, are small, narrow indentations, longer than wide, with a gap suitable for placement of a basket or net. Simple-gap fishways of similar size also occur. In contrast, in Bolivia the V-shaped features are much larger, with arms 30 m long on average and the width between arms even greater, constituting much larger outgrowths in the weir and often making the entire weir a zig-zag structure. Third, the surface of the capture area between two successive weirs is on average about twice as large in Bolivia as in Zambia. The larger surface area may have helped compensate for the much lower potential of the Bolivian system to concentrate fish. In the present-day Zambian system, fish are channelled into very small interception surfaces, the traps placed in fishways. In contrast, in the pre-Columbian system, fish are channelled into a large interception surface, the ponds of the capture area. (These become smaller, and fish become more concentrated [unless harvested, or eaten by non-human predators], as the dry season progresses). These morphological differences in the two weir-fishery landscapes show that despite their general convergence^27^, each of these independent cultural inventions also exhibits singularities.

These differences likely had important consequences for the ecological and social functioning of these two weir fisheries. The range of kinds of fish trapped in ponds in Bolivia may have been wider than in Zambia. Fish traps in Zambia selectively target migrating species, particularly juveniles. In addition to this ecological group, which is also well represented in Bolivian floodplains^27^ and would have been captured by the weir-pond system—out-migrating fish would have accumulated in ponds—ponds could provide habitat for non-migratory fish. These include small, air-breathing armoured catfishes (Callichthyidae, Loricariidae) that could have survived in ponds long into the dry season. They also include the *Synbranchus* swamp eel, an individual of which we found while excavating a pond in the dry season. *Synbranchus*, like some other fish species (e.g., the lungfish *Lepidosiren paradoxa*), can survive the dry season by estivating in moist sediments. Ponds would attract such fish at the beginning of the dry season and constitute reservoirs of these fish for people to harvest through much of the dry season. Although swamp eels are not consumed in the Llanos de Moxos today, they are the most abundant fish remains of archaeological dwellings in the region^7,36^. In Zambia, the efficient concentration of fish in traps set in fishways means that these traps have to be checked regularly and repeatedly to avoid damage or death (and rotting) of fish^34^. Once collected, fish have to be processed (e.g., smoked) for long-term conservation. Each day’s harvest is collected by individual fishermen and transported (usually in canoes) to sites where fish are prepared and consumed. In contrast, in Bolivia, fish were trapped in ponds, bodies of water large enough so that fish could have been stored alive there for some time, as Erickson^24–26^ has emphasized. How long into the dry season fish could be ‘live stored’ in ponds would depend on how fast ponds dry out. Fishing efficiency would increase as ponds dried out, but so would the intensity of competition between humans and other fish predators such as waterbirds^37,38^. (Piscivorous birds and crocodilians, along with other animals attracted by ponds, could also have become prey for humans.) The use of mosquito-net enclosures connected to fishways to accumulate and keep fish alive is a recent innovation in the Zambian fishery (Supplementary Fig. S8), indicating that it is advantageous to fishermen in this system as well to store fish alive and thereby reduce the frequency with which traps must be visited.

In the Bolivian system, fish were likely transported to residential sites in forest islands on foot along causeways, which are much more common than canals in our study site. As in pond-fishing in present-day Africa^39–42^, harvesting fish could have involved both individual and collective activities. The wide range of social organization through which pond fisheries can be exploited, and the flexibility they permit in organizing individuals’ activities over time, are well suited to societies in which fishing was only one component of a multi-activity subsistence system. The Zambian weir fishery, where daily harvesting of traps and nets is the rule, is more suited to the commercialization of dried fish.

Archaeology thus far offers few clues to the other subsistence activities the builders of the pre-Columbian fishery may have engaged in. Many other types of earthworks are known from different parts of the Llanos de Moxos. These include monumental mounds (large mounds with a probable political or spiritual function)^43,44^, man-made forest islands (elevated platforms that served as temporary settlements)^45^, shell mounds^46^, and agricultural raised fields of varying morphology^47^. All of these have yielded plant and animal remains documenting multi-activity subsistence systems, but none of these have so far been found in the area with the weir/pond fishery^48^. As already noted, causeways and canals, often several km long, occur in the area, indicating intense circulation of people, but these structures (like ring ditches, also found in the study region^49^) have so far yielded no clues about subsistence activities. The recent evidence that rice was domesticated by the mid-Holocene in a site about 100 km north of the study area^50^ suggests a key and hitherto completely overlooked role of agriculture based on a wetland-adapted crop.

**Figure 9:**
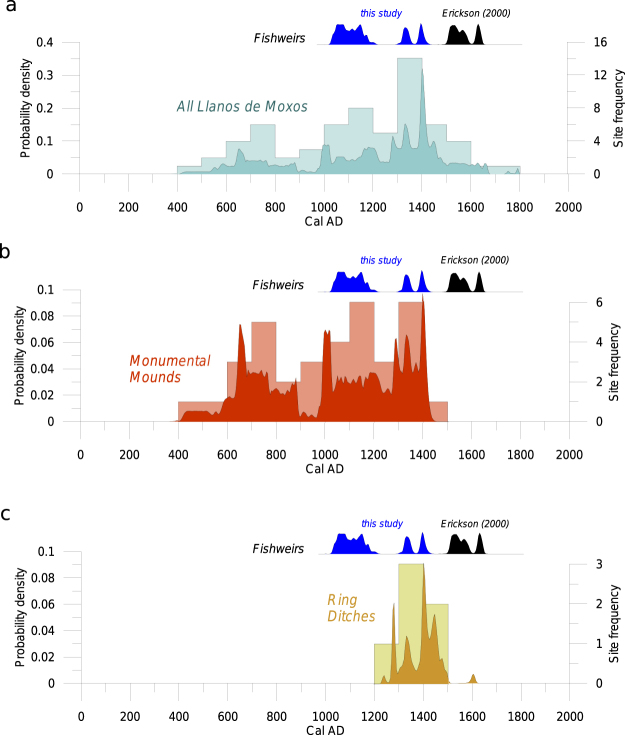
Sum of the probability distributions (SPDs) and frequency histograms for sites in the Llanos de Moxos over the last 2000 years in comparison with the calibrated dates for the San Joaquín fishery. (**a**) All types of archaeological sites; (**b**) Monumental mounds; (**c**) Ring ditches.

Radiocarbon dates obtained in the context of this study and from Erickson^24^ currently situate the fishery between Cal. AD 1030–1650, in the final centuries of pre-Columbian occupation of the Llanos de Moxos. A comparison with the available chronology for the region (Fig. 9) shows contemporaneity of the fishery both with the monumental mounds^43^ and the ring ditches^51^. A cultural association with the latter is more likely given their spatial proximity. Ring ditches have been found in forest islands within 10 km of the fishery (Fig. 1). The fishery precedes and follows construction of ring ditches (Fig. 9, Supplementary Table S3), showing that its use, whether continuous or interrupted, transcended the duration of particular archaeological cultures.

Ceramic fragments found in the pond sediments could not be dated. They bore no decorative motifs and were too small to allow identification of their style or cultural tradition. The ease with which we found them suggests that they are abundant in pond sediments, testifying to the use of ponds by humans.

Although pond fishing is documented throughout the world and water management was mastered by pre-Columbian populations in the Americas, this integrated system of extensive weirs concentrating fish into ponds seems to be unique. We are not aware of any other present-day, historical or archaeological example anywhere in the world. There is archaeological evidence for pre-Columbian pond fishing at Loma Salvatierra, about 150 km southwest of our study site, but weirs are absent there^52^. Natural or human-made ponds, known as *aguadas*, were used by the ancient Maya for year-round household water storage^53–55^, a feature particularly relevant in karstic regions such as those inhabited by the lowland Maya. There is no indication that *aguadas* were used for fish farming. In our study region in the Llanos de Moxos, storage of household water seems unlikely to have been an important function of floodplain ponds. Because settlements were probably located on the forest islands, having a few large ponds near forest islands would have made more sense than having a large number of small ponds scattered through the savannah. However, in addition to trapping fish in conjunction with weirs, ponds likely also functioned, as postulated by Erickson^24–26^, to hold water and thereby maintain alive fish captured by weirs, for some period after the water receded.

As noted above, ponds are absent in the present-day Zambian weir fishery. Other parts of Africa, however, offer the best-documented examples of the use of ponds to trap fish following seasonal flooding^39–42,56^. Pond fishing in Africa involves both artificial and natural ponds, the latter being usually modified to improve fish capture. However, none of the African pond-fishing systems makes use of extensive weirs to channel movements of fish. In Africa, other ways of concentrating fish in ponds are also observed, such as active feeding to attract fish to ponds^42,56^.

The patterns we see today are a snapshot of the state of these embankments at a particular time. Thorough archaeological study, including dating of different structures, would be essential to interpreting the cultural landscape. What techniques and tools were used to build weirs and dig ponds? What fishing practices were used to harvest fish in ponds? What fish species were harvested? Although information on the type of fish consumed is available through archaeological work in other parts of the Llanos de Moxos^7,52^, this information may not be transposable to the weir/pond fishery studied here because cultural practices may have varied greatly in space and time, particularly in relation to the type of environment exploited. Most importantly, how were fishing activities organized in space and time relative to other subsistence activities, notably agriculture? Did people farm in the floodplains (e.g., in pond margins), in forest islands, or both? Several considerations suggest that archaeological investigations in our study area would be highly fruitful. (i) Constructing and maintaining such an extensive network of weirs and ponds would have required social coordination, as proposed by Erickson^24^, suggesting occupation by sizable numbers of people organized in complex societies, a suggestion reinforced by the fact that the pre-Columbian Baures people built large towns^57^. (ii) Although we were not looking for them, we found potsherds with little effort while digging into a pond. (iii) Vestiges of pre-Columbian occupation sites are present in the forest islands of the site^24,49^ (authors’ personal observations). Archaeological investigation in our study area would reveal key features for refining our understanding of this compelling and unique cultural landscape.

## Methods

### Study site and sources of information

The study site is located *c*. 40 km ESE of the town of Baures, Beni, Bolivia (Fig. 1), and covers 155 km^2^ in a seasonally flooded savannah. Flood waters probably come from local precipitation and from the runoff from hills located to the south (locally called *monte de Guarayos*) (Supplementary Fig. S2). The landscape is a mosaic of seasonally flooded grassland (in a virtually flat basin ranging from 140 to 150 m asl in the study area) and forest islands on never-flooded higher-lying ground (160 to 170 m asl). Forest islands show remains of human settlements that were occupied until the European conquest^57^. Canals, causeways and earthen fish weirs (henceforth referred to simply as “weirs”) built by pre-Columbian populations cross the grassland, connecting forest islands^24,58^. Numerous ponds also occur in the grasslands. We mapped and analysed vestiges of weirs and ponds using three types of remote sensing data: (i) the World Imagery ArcGIS layer (Esri, DigitalGlobe, Earthstar Geographics, CNRS/Airbus DS, GeoEye, USDA FSA, USGS, Getmapping, Aerogrid, IGN, IGP, and the GIS User Community) at *c*. 1 m resolution; (ii) aerial photography from a small airplane (0.2 m resolution) and, mostly for illustration, (iii) photographs taken using a kite-borne camera flown at low altitude (1.2 cm resolution). We used PhotoScan (Agisoft) to map aerial photographs and build orthophotos, digital surface models and textural 3D models. A field trip conducted in October 2016 (end of the dry season) allowed ground-truthing—i.e., description of ground topography and vegetation, and measurement of heights of a subset of features–of 17% of the weirs, 26% of the V-shaped structures and 21% of the ponds that we investigated within the studied area (Fig. 1). We chose two sub-basins (Fig. 1) for a more detailed investigation of weirs, V-shaped structures and ponds, and their spatial associations, because the low level of woody vegetation cover in these sub-basins allowed a straightforward interpretation of the structures compared to other areas within the site (e.g., the area focused on by Erickson^24^). For each of the two sub-basins we estimated the range of the possible direction of water flow during the high-water season by combining information from interviews with a local resident, the topography of the sub-basin based on a digital elevation model provided by TerraSAR-X/TanDEM-X (Supplementary Fig. S2) and the azimuths of the diagonals of a rectangle encompassing the sub-basin. Throughout the paper we refer to this direction when classifying the orientation of objects in the landscape as “upstream” or “downstream” relative to other objects. Determining whether or not a visible feature is a weir is somewhat subjective, particularly in areas that have been heavily colonised by woody vegetation. We considered as weirs narrow ridges of raised earth showing angles that are interpreted as V-shaped structures. Some questionable structures were left unclassified. For this reason, our Fig. 1 depicts fewer weirs than can be seen in Erickson’s^24^ Fig. 4. Rectilinear ridges and troughs are considered causeways and canals, respectively.

### Quantifying patterns of spatial association between weirs and ponds in the sub-basins

To determine whether ponds and linear or zig-zag anthropogenic features (causeways, canals and weirs) are spatially associated, using aerial imagery we measured the distance between each pond and the nearest linear or zigzag anthropogenic feature. The observed distribution of distances revealed a decrease around 35 m, suggesting that this value could be used as a cut-off to consider whether a pond is associated (or not) with an anthropogenic feature. This value is compatible with our field observations. We then generated 10,000 random distributions of the same number of ponds within the sub-basins. To test for spatial association we computed the number of random distributions out of the 10,000 (equivalent to a p-value) that produced at least the same number of ponds observed within 35 m of the anthropogenic features.

As some ponds could have remained unnoticed on the aerial images (our study was conducted during the dry season, when all ponds were dry; ponds are identified from aerial images by vegetation differences, validated by ground-truthing [see below]), we estimated the degree of confidence supported by our analysis by computing the number of ponds that would have had to escape detection for the observed distribution of ponds not to differ from random expectation. We generated 100 random distributions of ponds for each number of a series increasing by increments of 10 starting from the observed number of ponds. For each set of 100 random distributions, we computed the number of distributions out of the 100 (equivalent to a p-value) that produced at least the same number of ponds observed within 35 m of an anthropogenic feature.

For those ponds found to be associated with weirs, we measured the distance between the centroid of the pond and the nearest V-shaped structure on the weir. Again, the observed distribution of distances revealed a decrease around 35 m, suggesting that this value could be used as a cut-off to consider whether or not a pond is associated with a V-shaped structure. This value is again compatible with our field observations. We then generated 10,000 random distributions of the same number of ponds along the weirs. To test for spatial association we computed the number of random distributions out of the 10,000 (equivalent to a p-value) that produced at least the same number of ponds observed within 35 m of a V-shaped structure along the weir.

### Characterising the physical features of V-shaped structures and ponds

Our field observations rapidly showed that whereas the vegetation of the seasonal floodplain in our site was almost exclusively grasses, wherever the soil surface was elevated above flood level, the vegetation was dominated by shrubs and trees (mostly palms). Distribution of woody vegetation could thus be used as a proxy, easily delimited on aerial images, of soil elevation. Vegetation reflectance is in fact the main feature that makes causeways, canals, weirs and ponds detectable in satellite imagery and aerial photography. After confirming the relationship between soil elevation and vegetation cover by ground-truthing (i.e., ground observation of topography and vegetation cover), we used the spatial distribution of the various types of vegetation cover in aerial images as a reliable and easily scored indicator of the distribution of elevated soil along V-shaped structures and around pond rims.

We determined whether there was any evidence for a gap at the point of V-shaped structures that could have been a fishway. For 13 V-shaped structures we measured in the field the vertical distance between a line extended horizontally from the highest soil level at the point of the V to the lowest spot within the arms of the structure. We classified the V-shaped structures according to the presence of associated ponds and their position relative to the structure. The orientation of the V-shaped structures that occurred in the sub-basins was measured as the azimuth from the middle of the wide opening of the arms of the V to the point of the V.

Because our field observations rapidly revealed that ponds showed distinctive features testifying to movement of soil from pond basins and deposition forming pond berms (see below), we investigated from remote sensing for each pond whether the berm surrounded it completely, or was present only along a portion of the rim, by measuring the angle of the rim covered by woody plants, and its extreme azimuths. To determine whether the berms followed a particular orientation, we measured the azimuth from the centre of the pond to the rim point with the highest soil level, inferred from the densest woody plant cover (as confirmed by ground-truthing). Our work was conducted in the dry season, and all ponds were empty, with no standing water. We estimated the current depth of the pond’s basin (N = 6) by measuring the vertical distance between a line extended horizontally from the highest point of the berm to the soil surface at the deepest spot in the pond.

In order to illustrate morphological differences of potential functional importance between V-shaped structures in this Bolivian pre-Columbian fishery and the present-day Zambian weir-based fishery that appears to be its closest modern analogue^27^, we measured the width and the length of 100 V-shaped features in each system taken randomly from 270 and 501 features in Bolivia and Zambia, respectively. Measurements were done on the World Imagery ArcGIS layer (*c*. 1 m resolution) for the Bolivian features and on satellite images taken by the Pléiades sensor (July 2013; 0.5 m resolution) for the Zambian features.

### Quantifying the potential of weirs and ponds to spatially concentrate fish resources

Both weirs and ponds function to concentrate fish from a large area of floodplain into a much smaller area, where they can be more easily captured. To estimate the relative ‘concentration factor’ we calculated the area of floodplain between each pair of adjacent weirs in the two focal sub-basins (15 inter-weir areas in total), and the ratio between the surface of this ‘capture area’ and the surface area of the collecting structures. The collecting structures of an inter-weir area were (i) the isolated ponds, (ii) the ponds associated with the weir marking the area’s downstream limit and (iii) the V-shaped structures of this weir that were not associated with an identified pond. Pond limits were defined based on vegetation differences seen from remote sensing and are thus approximate. For comparison, we computed the concentration potential of 15 inter-weir areas in the present-day fishery in Zambia. Traps fitted to fishways have entrance hoops 0.3 to 1.5 m in diameter^34^. We considered that each fishway represented a circular area of interception of 1 m in diameter. We computed the ratio between the surface of the ‘capture area’ and the total surface of interception by traps on fishways in the weir marking the capture area’s downstream limit.

### Statistical analyses

Statistical analyses were performed using R 3.3.2^59^, and the packages rgdal^60^, maptools^61^, rgeos^62^, sp^63^, circular^64^ and Directional^65^.

### Excavation of a trench and a quadrat in a pond

In one pond (S13.77415° W63.35571°), which was located just upstream of a V-shaped structure of a weir, we dug a 14-m-long trench, 1 m deep, 0.5 m wide, from the centre of the pond toward the point of the V. We identified three stratigraphic units which we sampled for geochemical analyses and radiocarbon dating. Two additional pits were dug: one in the floodplain outside the pond in order to have a reference profile for comparison of soil properties, and a 2.5 × 2.5 m 0.2 m deep quadrat to one side of the excavated trench in the pond basin to search for potsherds additional to those we had found while digging the trench. Descriptions of the horizons/units were carried out following the guidelines for soil description of the FAO^66^. Ceramic fragments and charcoal were found in the trench and in the quadrat (Fig. S7).

### Laboratory analysis

For the analysis of total elemental composition of particulate matter, samples were dried (105 °C) and ground to pass a 1-mm screen. The prepared samples were digested in an open vessel with concentrated hydrochloric and nitric acid (aqua regia). The elements dissolved in the acid were analysed by ICP-OES/ICP-MS at the NRM laboratories in Berkshire, UK. Radiocarbon analysis of a charcoal fragment and of the humin fraction of a soil bulk sediment sample, both taken from the base of Unit 2, was conducted at the Beta Analytic AMS laboratory in Florida and calibrated using BetaCal3.21^67^ and the SHcal13 calibration curve for the southern hemisphere^68^.

### Dating the time of use of the studied earthworks

In order to compare the dates obtained for the fishery with the regional chronology, we compiled all the available radiocarbon dates from the literature (see Supplementary Table S3). We present those dates through the sum of the probability distributions (SPDs)^69^. SPDs are a standard method for representing chronological trends in radiocarbon datasets. They are produced by calibrating each independent date in the sample and adding the results to produce a single density distribution. This has the advantage of including the full range of probabilities associated with calibrated dates, instead of using single point estimates^69,70^. Here, we produced SPDs for the whole Llanos de Moxos and for particular archaeological cultures in the vicinity of the fisheries using OxCal v4.2.4 and the ShCal13 calibration curve^68,71^. A binning procedure was applied to account for sites that had multiple dates within a phase^69,70,72^. Dates within sites were ordered, grouped within 100-year bins, and those occurring within the same bin were merged using the R_Combine function in OxCal. In addition to the SPDs, we present histograms of the number of occupied sites based on the medians of the calibrated dates per 100-year intervals (Fig. 9).

### Data availability

The datasets generated and/or analysed during the current study are available from the corresponding author on reasonable request.

## Electronic supplementary material

Supplementary Information

Supplementary Video 1
