# Supplementary material for: The unique functioning of a pre-Columbian Amazonian floodplain fishery
Source: Sci Rep. 2018 Apr 16;8:5998. doi: 10.1038/s41598-018-24454-4 (PMC5902550; doi:10.1038/s41598-018-24454-4)
Supplement: Supplementary file 1 — Supplementary Information [file 41598_2018_24454_MOESM1_ESM.pdf]

Supplementary material associated with:
Blatrix R., Roux B., Béarez P., Prestes-Carneiro G., Amaya M., Aramayo J. L., Rodrigues L., Lombardo U., Iriarte J., de Souza J. G., Robinson M., Bernard C., Pouilly M., Durécu M., Huchzermeyer C. F., Kalebe M., Ovando A., McKey D. The unique functioning of a pre-Columbian Amazonian floodplain fishery. *Scientific Reports* (2018).

Supplementary elements of discussion

Spatially explicit quantitative analysis of landscape features using remote sensing and ground-truthing allowed us to draw a rough outline of the functioning of the pre-Columbian weir fishery in the Baures region of the Llanos de Moxos, and to highlight some of its specificities in comparison with its closest analogue, the present-day weir fishery in Zambia. However, while our study has provided some answers, it also opens new questions. The precise mechanisms by which V's channel fish into ponds are still unresolved. If these ancient earthworks still function, even if imperfectly, to channel the movement of water and of fish, field studies could be devised to test the hypotheses we propose.

Alternative explanations for the specific orientation in the distribution of woody vegetation around ponds

Erickson[1] contended that ponds were dug by humans. If so, deposition of excavated soil only on the downstream shore would certainly have made ponds better fish traps. However, an alternative hypothesis for the distributions around ponds of berms, woody vegetation or both, is that they were shaped by scouring due to the water flow. Water flowing around an obstacle such as a tree or shrub (fallen or standing) can create a vertical vortex upstream of the obstacle that leads eventually to the formation of a scour, the scoured material being then deposited downstream, forming an accretion[2]. Supplementary Fig. S9 shows ponds that were likely formed in this way in the San Martín floodplain, just 30 km north of our study area. The ponds have a 'forest hump' on higher ground just downstream of the pond in the shape of a sediment plume. The digital elevation model, and the topographic profile, show that the formation of these sediment plumes downstream of flow obstacles also happens at the scale of the large forest island. These sediment plumes are oriented following the direction of the water flow, as shown in the insets B and C of Supplementary Fig. S9. This is consistent with a spatial analysis of pond distribution and orientation within a larger area of the San Martín floodplain[3]. Just behind the obstacle the sedimentary plume is more elevated and, therefore, covered with trees. Moving downstream along the sedimentary plume, the elevation is reduced and the vegetation cover changes to shrubs, then to savannah. Further field studies are needed in order to assess whether this occurred only during flood regimes of the past, or whether flood regimes today continue to drive such processes.

Prolonged gradual formation of a scour and accretion, and one-time rapid excavation of a basin by people who deposited the material just downstream, should produce very different vertical profiles in the berm, offering a way to distinguish between hypotheses about the natural or cultural origin of ponds. Nevertheless, a natural origin of ponds would not exclude the possibility that the ponds were later used, managed and modified by pre-Columbian inhabitants.

Detailed interpretation of the functioning of the integrated system of weirs and ponds in Bolivia

In most weir fisheries, how fish are trapped can be explained by relatively simple hydraulic principles: fish following (actively or passively) a current of flowing water are constrained by a barrier and driven to a relatively narrow gap where a trapping device is placed. The present-day Zambian weir-based fishery functions in this way: out-migrating fish that encounter a weir move along it until they reach a gap (often V-shaped) where the current drives them into a net or trap. Without fishways at the point of V's on the Bolivian weirs, no current would have been generated through the V's. Thus the force that in most kinds of weirs drives fish to and through fish-concentrating structures, such as V's, could not have acted here.

Given these results, we have been unable to conceive of any mode of functioning of the Bolivian system that relies purely on hydraulics. However, fish that migrate in and out of seasonal floodplains are not simply passive drifters that flow with the current; their movements are also driven by behavioural preferences[4,5]. Fish that migrate seasonally in and out of floodplains use current as a cue and they are capable of detecting very small differences[4,5]. They likely use higher flow velocity of outflowing water as a cue to guide them to permanent water, and slow flow velocity, along with water depth, as a cue that a permanent water body has been reached. We propose a hypothesis for the functioning of the integrated weir/pond system that relies not simply on hydraulics, but on the interactions between hydraulics and fish behavioural preferences.

As in the Zambian weir fishery (authors' personal observations), weirs were likely completely submerged at highest water level. The absence of fishway gaps thus posed no barrier to in-migrating fish. Again as in the Zambian fishery, during the first stage of flood recession enough fish must have escaped in water flowing over the weirs to allow persistence of fish populations. However, the fish most frequently captured by weirs in the Zambian system are benthic species, juvenile catfish and mormyrids, that tend to hug the bottom during out-migration. Similar behaviour can be expected of neotropical floodplain-breeding fish such as small catfish (Pimelodidae, Doradidae, Callichthyidae, and others) and Synbranchidae. When they reached a weir, these fish would have tended to move along the weir, following the current downstream until reaching a deeper water body (the pond), where they would have remained, particularly if they perceived no further (or slower) downstream current. The fact that V's are higher than other parts of the weir may have reduced the current behind the V relative to either side of the V. Also, in low-gradient floodplains such as this one, microtopography is not the only factor affecting variation in flow velocity: vegetation offers resistance to water flow[6]. The more abundant vegetation in and around the ponds could have contributed to lower flow velocity in the ponds than in areas to either side. The shade and food resources provided by denser vegetation in and around ponds would also have contributed to their attractiveness (as in African pond fisheries[7,8]).

Later, when water level became so low that outflow was completely blocked even by the lowest parts of the weirs, and as water evaporated and/or seeped into the soil, any fish trapped in the inter-weir areas would similarly seek out the deepest water and congregate in ponds. As the ponds dried, fish were increasingly concentrated there, where they could be easily harvested by diverse methods[7,9-11].

Our data thus suggest that, like the builders of the estuarine fishery described by Greene *et al.*[12], the builders of the floodplain weir system in the Baures region did not just use simple hydraulic forces of the inflowing and outflowing water to trap fish, they also must have been keen observers of the behaviour of migrating fish, exploiting their knowledge to make an ingenious fish-trapping system.

Our hypotheses attempt to account for the most common patterns in the shape and spatial association of these earthworks. However, we also document other variants. First, although most ponds are upstream of a V-shaped structure, some are downstream of such structures, suggesting that the technique of construction and use may have varied among individuals or across time. Another kind of variation observed is the presence of ponds along causeways. Many of these ponds may have simply been borrow pits from the digging of earth to build the causeways, but several others clearly interrupt the causeway and are associated with a downstream berm, suggesting they also were designed as fish-trapping features. Other ponds along causeways are clearly associated with a V-shaped structure, suggesting that causeways may also have doubled as fish weirs. Whether causeways served the two functions, transport and fish-trapping, simultaneously or successively remains unclear.

Earthen archaeological vestiges and non-human soil engineers

Archaeological remains of earthworks sometimes erode rapidly. However, although the fishery based on earthen weirs in the Baures region has been abandoned for at least 300 years[1], vestiges of the weirs are still visible today. Today, these vestiges are mostly comprised of accumulated earthworm casts, along with a few termitaria, colonized by plants that are taller (and a greater proportion of woody plants) than in the rest of the floodplain. Soil organisms took advantage of spatial micro-heterogeneity created by humans, colonizing the well-drained soils of elevated structures, stabilizing these soils against erosion by their production of biostructures and thereby maintaining topographic heterogeneity through time, as for other legacies of pre-Columbian engineering activities such as raised fields[13-15]. Soil organisms disturb stratigraphy, sometimes making it difficult to date archaeological deposits and study their vertical structure[16,17], but on the other hand, the actions of soil organisms have been essential for preserving the physical vestiges of earthworks, allowing their detection and the description of their spatial organization.

Supplementary Fig. S1: Aerial views of vestiges of pre-European earthworks. (a) Vestige of a causeway colonised by palm trees, connecting two forest islands. (b) Vestige of a fish weir, in the foreground, showing successive V-shaped structures, a causeway colonised by palm trees in the right background, and a pond in between. Photos (17 October 2016): Bruno Roux.

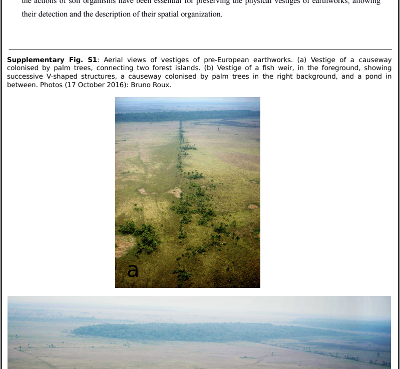

Supplementary Fig. S2: (a) Digital elevation model (srtm) at the regional scale around the study area. The study area is located within the basin of the small river San Martín, and is disconnected from the Guaporé/Itenez basin by an orographic barrier. Flood waters in the study area probably come from local precipitation and from the runoff from hills located to the south (locally called monte de Guarayo). As a result, waters flows consistently to the NNW throughout the wet season. Fish resources are those from the San Martín basin. In-migrating fish reach the study area probably only when the San Martín basin is filled enough for the standing water body to encompass the study area. At the end of the high water season, out-migrating fish follow the NNW flow of the receding water. (b) Digital elevation model (at *c.* 12 m resolution, provided by TerraSAR-X / TanDEM-X) of the study area showing that the basin is somewhat lower in altitude in the north-west than in the south-east. According to the digital elevation model, water in the studied sub-basins flows to the northwest during the high-water season (arrows). This is in agreement with the account from a local informant. The area studied by Erickson[1], delimited by the dashed white line, is substantially higher than our study area (plain white polygon), explaining why the former has been overgrown by shrubs and trees. © DLR (2017).

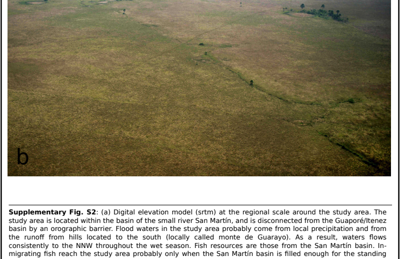

Supplementary Fig. S3: Digital surface model and oblique views of textural 3D models of a weir reconstructed by stereophotogrammetry, using series of photographs taken with a kite-borne camera. Two V-shaped structures are visible along the weir. A large pond is visible at the bottom of the picture, away from the weir, and probably not associated with it.

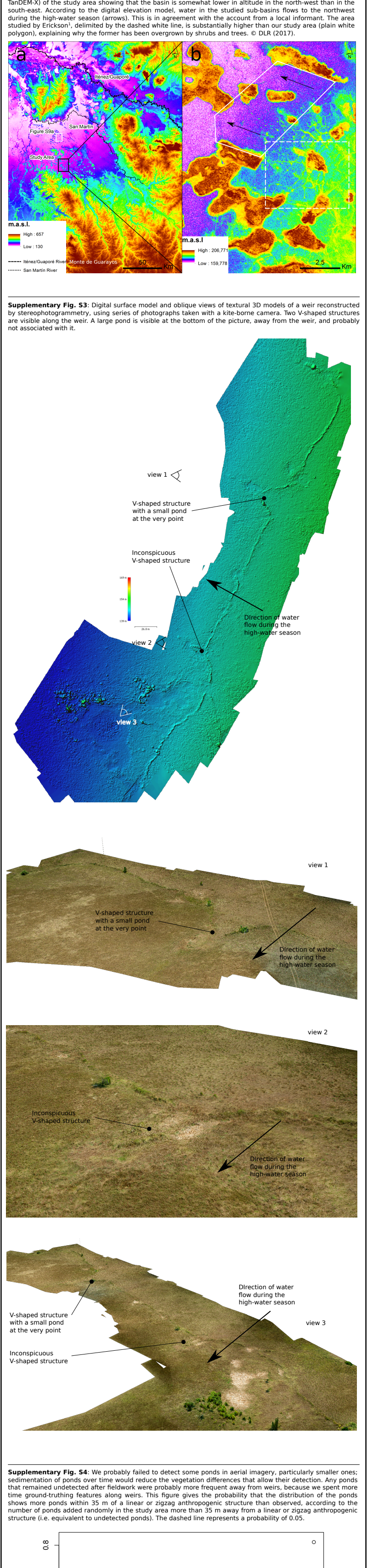

Supplementary Fig. S4: We probably failed to detect some ponds in aerial imagery, particularly smaller ones; sedimentation of ponds over time would reduce the vegetation differences that allow their detection. Any ponds that remained undetected after fieldwork were probably more frequent away from weirs, because we spent more time ground-truthing features along weirs. This figure gives the probability that the distribution of the ponds shows more ponds within 35 m of a linear or zigzag anthropogenic structure than observed, according to the number of ponds added randomly in the study area more than 35 m away from a linear or zigzag anthropogenic structure (i.e. equivalent to undetected ponds). The dashed line represents a probability of 0.05.

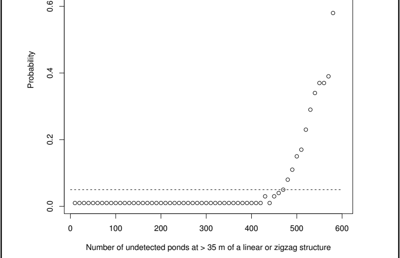

Supplementary Fig. S5: Aerial view of a V-shaped structure not associated with a pond. Photo (11 October 2016): Bruno Roux.

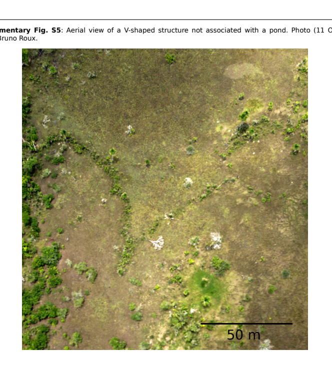

Supplementary Fig. S6: Digital surface model and oblique view of a textural 3D model of a pond along a causeway reconstructed by stereophotogrammetry, using series of photographs taken with a kite-borne camera. The downstream side of the pond has a berm colonized by trees.

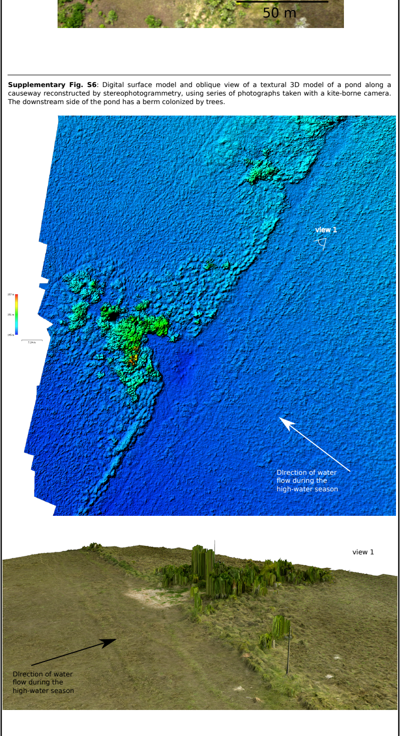

Supplementary Fig. S7: Map of the trench and the quadrat excavated in a pond associated with a V-shaped structure and photos of some of the pottery sherds found while digging. Photos: Rumsaïs Blatrix.

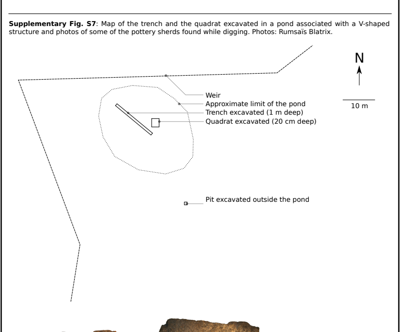

Supplementary Fig. S8: A mosquito-net enclosure connected to a fishway in a weir of the Bangweulu Basin, Zambia, designed to accumulate and keep fish alive, reducing the need to check traps daily. Photo: Carl F. Huchzermeyer.

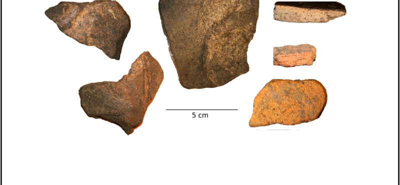

Supplementary Fig. S9: The digital elevation model (provided by TerraSAR-X / TanDEM-X) shows that the orientation of the berms associated with the ponds (panels b and c) is consistent with that of the large berms (sediment plumes shown in panel a) deposited downstream of large forest islands. The same depositional mechanism acts at both scales, that of small ponds and that of large forest islands. The topographic profiles (panels d and e) go across a sedimentary plume formed downstream of a large forest island. This is about 1 meter high right behind the forest island, and about 20-40 cm high 2 km further downstream. The area depicted in panel a is located 30km north of our study area (plain white rectangle in Figure S2a). © DLR (2017).

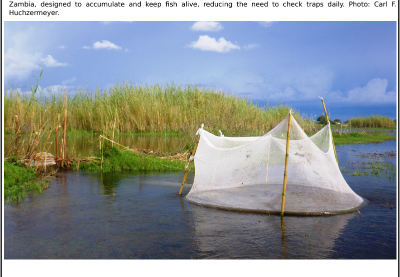

Supplementary Video 1: Panning from right to left, one sees first the weir (extending in a straight line across the floodplain), then the right arm of the V, which angles off downstream of the pond to join the pond's berm, which is marked by dense woody vegetation. The left arm of the V then comes back to join the weir, which continues across the floodplain in the same orientation as before its interruption by the V and associated pond.

Supplementary Table S1: AMS radiocarbon ages of charcoal and soil bulk samples, given both as 14C age BP and calibrated radiocarbon age in BP and AD format at two-sigma level. We chose to date the humin fraction (residues) because in general this fraction is considered to be more stable and less affected by contamination[18].

Profile	A	B
Depth (cm)	85	80
Material<br>Dated fraction	Bulk<br>Humin (RES)	Charred Material
14C age	620 ± 30 BP	970 +/- 30 BP
95,4 % (2σ) cal age ranges	(49,9%) 1310 - 1360 cal AD<br>(640 - 590 cal BP)<br>(45,5%) 1378 - 1424 cal AD<br>(572 - 526 cal BP)	1030- 1180 cal AD<br>(920-770 cal BP)
Lab Number	Beta - 478379	Beta - 478379

Supplementary Table S2: Down-profile values of selected geochemical parameters and Chemical Index of Alteration (CIA) of profiles A, B and C in the pond and the reference profile (see Fig. 8). The Chemical Index of Alteration[19], an index of weathering, was calculated as follows: CIA = 100 x Al2O3 / (Al2O3 + CaO + Na2O + K2O).

Elemental composition

Depth cm:	Na_{total}	Mg_{total}	K_{total}	Ca_{total}	Al_{total}	P_{total}	Fe_{total}	CIA	C_{total}	N
**Profile A**										
Unit 1, 70	71.2	340	586	1298	67032	194	5662	98.0	4.32	0.509
Unit 2, 85	58.5	327	505	1077	57903	84	5702	98.0	2.62	0.399
Unit 3, 90	66.8	459	646	622	76537	<50	9189	98.8	0.44	0.186
**Profile B**										
Unit 1, 65	71.7	393	658	901	77225	100	7121	98.5	2.52	0.347
Unit 1, 80	59.1	331	545	619	62597	57	6547	98.7	1.47	0.238
Unit 1, 90	57	399	658	518	69059	<50	7650	98.9	0.41	0.137
**Profile C**										
Unit 1, 55	64.3	389	624	784	75025	62	6989	98.7	1.39	0.216
Unit 2, 85	69.1	427	629	731	78144	53	8425	98.7	1.13	0.184
Unit 3, 95	44.4	385	496	452	60777	<50	8765	98.9	0.28	0.111
**Reference profile**										
**10-20**	83.5	373	793	548	80260	122	7953	98.8	2.27	0.295
**30-40**	50	364	616	629	59828	<50	12597	98.5	0.45	0.125

Supplementary Table S3: Review of the published radiocarbon dating of archaeological sites in the Llanos de Mojos.

Site name	14C yr BP	Lab number	Reference	Latitude	Longitude
Bella Vista-1	2980 ± 44	Erl-6559	20	-13.2635	-63.7066
Bella Vista-1	726 ± 41	Erl-6558	20	-13.2635	-63.7066
Bella Vista-1	634 ± 44	Erl-6561	20	-13.2635	-63.7066
Bella Vista-1	568 ± 43	Erl-6560	20	-13.2635	-63.7066
Bella Vista-2	607 ± 28	KIA-38833	21	-13.2635	-63.7066
Bella Vista-2	775 ± 25	KIA-48489	21	-13.2635	-63.7066
Bella Vista-2	782 ± 27	KIA-38831	21	-13.2635	-63.7066
Bella Vista-2	783 ± 25	KIA-48488	21	-13.2635	-63.7066
Bermeo, San Ignacio	1340 ± 100	FFM a	22	-14.895	-65.377
Bermeo, San Ignacio	1085 ± 35	Poz-36133	22	-14.895	-65.377
Bermeo, San Ignacio	905 ± 30	Poz-36131	22	-14.895	-65.377
Bermeo, San Ignacio	695 ± 30	Poz-36130	22	-14.895	-65.377
Bermeo, San Ignacio	585 ± 30	Poz-39568	22	-14.895	-65.377
Bermeo, San Ignacio	465 ± 35	CAM a	22	-14.895	-65.377
Bermeo, San Ignacio	355 ± 25	FFM a	22	-14.895	-65.377
El Cerro	620 ± 40	AA30390	23	-13.23447	-65.4123
El Cerro	620 ± 40	AA30391	23	-13.23447	-65.4123
El Cerro	610 ± 70	117220	23	-13.23447	-65.4123
El Cerro	600 ± 45	AA30392	23	-13.23447	-65.4123
El Cerro	570 ± 45	AA30389	23	-13.23447	-65.4123
El Cerro	535 ± 45	AA30388	23	-13.23447	-65.4123
El Cerro	480 ± 50	117218	23	-13.23447	-65.4123
El Cerro	470 ± 90	117221	23	-13.23447	-65.4123
El Progreso, San Borja	790 ± 70	MAM	24	-14.825	-66.713
El Progreso, San Borja	635 ± 55	MAM	24	-14.825	-66.713
Filones	294 ± 22		25	-14	-65.7
Filones	395 ± 77		25	-14	-65.7
Filones	200 ± 30		25	-14	-65.7
Filones	930 ± 35		25	-14	-65.7
Isla Rodeo, loma pequeña	915 ± 25	N/A	1	-15.11	-65.49
Jasiaquiri	444 ± 25	KIA-48486	21	-13.7214	-63.7411
Jasiaquiri	500 ± 25	KIA-48484	21	-13.7214	-63.7411
Jasiaquiri	596 ± 25	KIA-48487	21	-13.7214	-63.7411
Jasiaquiri	610 ± 25	KIA-48482	21	-13.7214	-63.7411
Loma Alta de Casarabe	1400 ± 75	SI-5371	26	-14.93	-64.29
Loma Alta de Casarabe	1375 ± 60	SI-5375	26	-14.93	-64.29
Loma Alta de Casarabe	1315 ± 70	SI-5372	26	-14.93	-64.29
Loma Alta de Casarabe	1140 ± 90	SI-5384	26	-14.93	-64.29
Loma Alta de Casarabe	1060 ± 65	SI-5382	26	-14.93	-64.29
Loma Alta de Casarabe	1055 ± 55	SI-5870	26	-14.93	-64.29
Loma Alta de Casarabe	900 ± 70	SI-5380	26	-14.93	-64.29
Loma Alta de Casarabe	835 ± 70	SI-5381	26	-14.93	-64.29
Loma Alta de Casarabe	830 ± 70	SI-5383	26	-14.93	-64.29
Loma Alta de Casarabe	755 ± 95	SI-5385	26	-14.93	-64.29
Loma Alta de Casarabe	680 ± 120	SI-5373	26	-14.93	-64.29
Loma Esperanza II, Río Apere	1030 ± 45	N/A	1	-14.83	-65.47
Loma Esperanza II, Río Apere	600 ± 25	N/A	1	-14.83	-65.47
Loma Kiusíu	1355 ± 60	SI-4442	26	-14.74	-65.06
Loma Kiusíu	920 ± 70	SI-4438	26	-14.74	-65.06
Loma Kiusíu	895 ± 60	SI-4437	26	-14.74	-65.06
Loma Kiusíu	870 ± 70	SI-4440	26	-14.74	-65.06
Loma Kiusíu	820 ± 60	SI-4439	26	-14.74	-65.06
Loma Kiusíu	690 ± 65	SI-4436	26	-14.74	-65.06
Loma Mary	1360 ± 60	SI-4118	26	-14.81	-65
Loma Mary	970 ± 80	SI-4116	26	-14.81	-65
Loma Mary	940 ± 70	SI-4115	26	-14.81	-65
Loma Mary	640 ± 60	SI-4114	26	-14.81	-65
Loma Mendoza	2800 ± 29	Bln-5328	27	-14.8817	-64.4522
Loma Mendoza	2244 ± 54	Erl-5610	27	-14.8817	-64.4522
Loma Mendoza	1557 ± 39	Erl-4789	27	-14.8817	-64.4522
Loma Mendoza	1512 ± 54	Erl-5613	27	-14.8817	-64.4522
Loma Mendoza	1508 ± 51	Erl-5604	27	-14.8817	-64.4522
Loma Mendoza	1465 ± 53	Erl-5606	27	-14.8817	-64.4522
Loma Mendoza	1443 ± 38	Erl-4787	27	-14.8817	-64.4522
Loma Mendoza	1417 ± 36	Erl-4790	27	-14.8817	-64.4522
Loma Mendoza	1410 ± 53	Erl-5609	27	-14.8817	-64.4522
Loma Mendoza	1407 ± 55	Erl-5608	27	-14.8817	-64.4522
Loma Mendoza	1407 ± 52	Erl-5611	27	-14.8817	-64.4522
Loma Mendoza	1371 ± 49	Erl-4786	27	-14.8817	-64.4522
Loma Mendoza	1357 ± 51	Erl-5603	27	-14.8817	-64.4522
Loma Mendoza	1342 ± 60	Erl-5607	27	-14.8817	-64.4522
Loma Mendoza	1338 ± 51	Erl-5605	27	-14.8817	-64.4522
Loma Mendoza	1316 ± 57	Erl-5616	27	-14.8817	-64.4522
Loma Mendoza	1313 ± 52	Erl-5612	27	-14.8817	-64.4522
Loma Mendoza	1296 ± 36	Erl-4788	27	-14.8817	-64.4522
Loma Mendoza	1254 ± 40	Erl-3207	27	-14.8817	-64.4522
Loma Mendoza	1253 ± 36	Erl-3205	27	-14.8817	-64.4522
Loma Mendoza	1238 ± 53	Erl-5615	27	-14.8817	-64.4522
Loma Mendoza	1235 ± 53	Erl-5614	27	-14.8817	-64.4522
Loma Mendoza	1231 ± 38	Erl-3206	27	-14.8817	-64.4522
Loma Mendoza	1186 ± 66	Erl-3202	27	-14.8817	-64.4522
Loma Mendoza	1134 ± 33	Bln-5327	27	-14.8817	-64.4522
Loma Mendoza	1077 ± 28	Bln-5325	27	-14.8817	-64.4522
Loma Mendoza	1065 ± 29	Bln-5214	27	-14.8817	-64.4522
Loma Mendoza	1050 ± 31	Bln-5212	27	-14.8817	-64.4522
Loma Mendoza	1043 ± 43	Erl-3201	27	-14.8817	-64.4522
Loma Mendoza	1036 ± 39	Bln-5344	27	-14.8817	-64.4522
Loma Mendoza	1030 ± 28	Bln-5211	27	-14.8817	-64.4522
Loma Mendoza	1026 ± 43	Erl-3208	27	-14.8817	-64.4522
Loma Mendoza	973 ± 45	Erl-3200	27	-14.8817	-64.4522
Loma Mendoza	961 ± 24	Bln-5345	27	-14.8817	-64.4522
Loma Mendoza	953 ± 38	Erl-3203	27	-14.8817	-64.4522
Loma Mendoza	950 ± 29	Bln-5326	27	-14.8817	-64.4522
Loma Mendoza	948 ± 29	Bln-5213	27	-14.8817	-64.4522
Loma Mendoza	651 ± 34	Erl-4791	27	-14.8817	-64.4522
Loma Mendoza	631 ± 29	Bln-5210	27	-14.8817	-64.4522
Loma Mendoza	609 ± 46	Erl-3199	27	-14.8817	-64.4522
Loma Palmazola	740 ± 60	SI-4048	26	-15.04	-64.8
Loma Palmazola	675 ± 70	SI-4113	26	-15.04	-64.8
Loma Salvatierra	3319 ± 42	Bln-5946LI	27	-14.8844	-64.4875
Loma Salvatierra	2594 ± 49	Erl-8011	27	-14.8844	-64.4875
Loma Salvatierra	1623 ± 30	KIA-31856	27	-14.8844	-64.4875
Loma Salvatierra	1489 ± 27	KIA-32720	27	-14.8844	-64.4875
Loma Salvatierra	1389 ± 30	Bln-5945-L	27	-14.8844	-64.4875
Loma Salvatierra	1380 ± 65	SI-5391	26	-14.8844	-64.4875
Loma Salvatierra	1350 ± 20	KIA-31854	27	-14.8844	-64.4875
Loma Salvatierra	1341 ± 24	KIA-31855	27	-14.8844	-64.4875
Loma Salvatierra	1337 ± 39	Bln-5860	27	-14.8844	-64.4875
Loma Salvatierra	1335 ± 26	KIA-38804	27	-14.8844	-64.4875
Loma Salvatierra	1325 ± 50	SI-5393	26	-14.8844	-64.4875
Loma Salvatierra	1316 ± 27	KIA-38803	28	-14.8844	-64.4875
Loma Salvatierra	1311 ± 26	Bln-5861	27	-14.8844	-64.4875
Loma Salvatierra	1300 ± 23	KIA-38805	28	-14.8844	-64.4875
Loma Salvatierra	1278 ± 53	Erl-8016	27	-14.8844	-64.4875
Loma Salvatierra	1275 ± 39	Bln-5947 LI	27	-14.8844	-64.4875
Loma Salvatierra	1275 ± 46	Erl-8014	27	-14.8844	-64.4875
Loma Salvatierra	1240 ± 28	KIA-38802	27	-14.8844	-64.4875
Loma Salvatierra	1239 ± 31	KIA-32718	27	-14.8844	-64.4875
Loma Salvatierra	1228 ± 23	KIA-38813	28	-14.8844	-64.4875
Loma Salvatierra	1224 ± 24	KIA-38808	28	-14.8844	-64.4875
Loma Salvatierra	1223 ± 26	KIA-38812	28	-14.8844	-64.4875
Loma Salvatierra	1201 ± 24	KIA-32719	27	-14.8844	-64.4875
Loma Salvatierra	1156 ± 25	KIA-31858	27	-14.8844	-64.4875
Loma Salvatierra	1105 ± 80	SI-5394	26	-14.8844	-64.4875
Loma Salvatierra	1060 ± 25	KIA-38800	27	-14.8844	-64.4875
Loma Salvatierra	1044 ± 44	Erl-8012	27	-14.8844	-64.4875
Loma Salvatierra	1041 ± 20	KIA-31859	27	-14.8844	-64.4875
Loma Salvatierra	1040 ± 45	SI-5878	26	-14.8844	-64.4875
Loma Salvatierra	1035 ± 95	SI-5390	26	-14.8844	-64.4875
Loma Salvatierra	1030 ± 44	Erl-8013	27	-14.8844	-64.4875
Loma Salvatierra	1007 ± 22	KIA-38810	28	-14.8844	-64.4875
Loma Salvatierra	1005 ± 50	SI-5879	26	-14.8844	-64.4875
Loma Salvatierra	985 ± 45	Erl-8015	27	-14.8844	-64.4875
Loma Salvatierra	957 ± 23	KIA-38801	27	-14.8844	-64.4875
Loma Salvatierra	956 ± 25	KIA-31857	27	-14.8844	-64.4875
Loma Salvatierra	948 ± 25	KIA-32717	27	-14.8844	-64.4875
Loma Salvatierra	947 ± 21	KIA-38807	28	-14.8844	-64.4875
Loma Salvatierra	932 ± 22	KIA-38809	28	-14.8844	-64.4875
Loma Salvatierra	896 ± 32	KIA-38814	28	-14.8844	-64.4875
Loma Salvatierra	820 ± 70	SI-5392	26	-14.8844	-64.4875
Loma Salvatierra	740 ± 23	KIA-38811	28	-14.8844	-64.4875
Loma Salvatierra	596 ± 22	KIA-38806	28	-14.8844	-64.4875
Moxos - Baures	335 ± 20	OS-17293	1	-13.65	-63.69
Río Iruyañez	620 ± 40	AA30390	29	-13.47	-65.73
Río Iruyañez	620 ± 40	AA30391	29	-13.47	-65.73
Río Iruyañez	610 ± 70	Beta-117220	29	-13.47	-65.73
Río Iruyañez	600 ± 45	AA30392	29	-13.47	-65.73
Río Iruyañez	570 ± 45	AA30389	29	-13.47	-65.73
Río Iruyañez	470 ± 90	Beta-117221	29	-13.47	-65.73
San Borja, Estancia Suárez	2830 ± 260	N/A	27	-14.9	-66.74
San Borja, Estancia Suárez	980 ± 170	N/A	27	-14.9	-66.74
San Borja, Estancia Suárez	600 ± 60	N/A	27	-14.9	-66.74
San Juan	5825 ± 70	AA30387	23	-13.32619	-65.4868
San Juan	5740 ± 40	117222	23	-13.32619	-65.4868
San Juan	1560 ± 45	AA30385	23	-13.32619	-65.4868
San Juan	1560 ± 50	117223	23	-13.32619	-65.4868
San Juan	1550 ± 45	AA30386	23	-13.32619	-65.4868
San Juan	1530 ± 45	AA30384	23	-13.32619	-65.4868
San Juan	1475 ± 55	AA3083	23	-13.32619	-65.4868
SM1	9420 ± 50	Poz-36135	30	-14.788163	-64.644784
SM1	9270 ± 60	Poz-34301	30	-14.788163	-64.644784
SM1	5800 ± 35	Poz-36136	30	-14.788163	-64.644784
SM1	5520 ± 40	Poz-22902	30	-14.788163	-64.644784
SM1	5520 ± 40	Poz-34231	30	-14.788163	-64.644784
SM1	5505 ± 35	Poz-24634	30	-14.788163	-64.644784
SM1	5460 ± 40	Poz-34232	30	-14.788163	-64.644784
SM1	5360 ± 40	Poz-24633	30	-14.788163	-64.644784
SM1	4945 ± 35	Poz-34230	30	-14.788163	-64.644784
SM1	4480 ± 40	Poz-58856	30	-14.788163	-64.644784
SM1	4415 ± 35	Poz-28855	30	-14.788163	-64.644784
SM1	3895 ± 35	Poz-34229	30	-14.788163	-64.644784
SM1	3830 ± 50	Poz-28854	30	-14.788163	-64.644784
SM1	345 ± 25	Poz-34228	30	-14.788163	-64.644784
SM2	5500 ± 40	Poz-38852	30	-15.166254	-65.042352
SM2	5380 ± 40	Poz-38851	30	-15.166254	-65.042352
SM2	4950 ± 40	Poz-38853	30	-15.166254	-65.042352
SM2	4770 ± 60	Poz-38850	30	-15.166254	-65.042352
SM3	7860 ± 50	Poz-38865	30	-14.727075	-64.67637
SM3	7790 ± 80	Poz-38866	30	-14.727075	-64.67637
SM3	5140 ± 40	Poz-38862	30	-14.727075	-64.67637

References for supplementary material

1. Erickson, C. L. An artificial landscape-scale fishery in the Bolivian Amazon. *Nature* **408,** 190–193 (2000).
2. Euler, T. & Herget, J. Controls on local scour and deposition induced by obstacles in fluvial environments. *Catena* **91**, 35-46 (2012).
3. Sigel, M. *Analysis of the geomorphology in northeast Bolivia with ArcMap* (BSc Thesis, University of Berne, Germany, 2012).
4. Feist, B. E. & Anderson, J. J. *Review of fish behavior relevant to fish guidance systems. University of Washington* (Report FRI-UW-9102, University of Washington, Fisheries Research Unit, 1991).
5. Espírito-Santo, H. M. V., Rodríguez, M. A. & Zuanon, J. Strategies to avoid the trap: stream fish use fine-scale hydrological cues to move between the stream channel and temporary pools. *Hydrobiologia* **792,** 183–194 (2017).
6. Harvey, J. W. *et al.* Hydroecological factors governing surface water flow on a low-gradient floodplain. *Water Resour. Res.* **45,** W03421 (2009).
7. Welcomme, R. L. A description of certain indigenous fishing methods from Southern Dahomey. *Afr. J. Trop. Hydrobiol. Fish.* **1,** 129–140 (1971).
8. Hauber, M. E., Bierbach, D. & Linsenmair, K. E. The traditional whedo aquaculture system in Northern Benin. *J. Appl. Aquac.* **23,** 67–84 (2011).
9. Craig, J. F., Halls, A. S., Barr, J. J. F. & Bean, C. W. The Bangladesh floodplain fisheries. *Fish. Res.* **66,** 271–286 (2004).
10. Renard-Toumi, A. & de la Croix, K. Les mares des plaines alluviales du Niger supérieur. *Dyn. Environnementales - J. Int. Géosciences Environ.* **32,** 14–35 (2013).
11. Comptour, M., Caillon, S. & McKey, D. Pond fishing in the Congolese cuvette: a story of fishermen, animals, and water spirits. *Rev. d'Ethnoécologie* **10** (2016).
12. Greene, N. A., McGee, D. C. & Heitzmann, R. J. The Comox harbour fish trap complex: a large-scale, technologically sophisticated intertidal fishery from British Columbia. *Can. J. Archaeol.* **39,** 161–212 (2015).
13. McKey, D. *et al.* Pre-Columbian agricultural landscapes, ecosystem engineers, and self-organized patchiness in Amazonia. *Proc. Natl. Acad. Sci. U. S. A.* **107,** 7823–7828 (2010).
14. Huchzermeyer, C. F. Fish and fisheries of Bangweulu wetlands, Zambia. (MSc thesis, Rhodes University, 2013).
15. Renard, D. *et al.* Ancient human agricultural practices can promote activities of contemporary non-human soil ecosystem engineers: a case study in coastal savannas of French Guiana. *Soil Biol. Biochem.* **62,** 46–56 (2013).
16. Stein, J. K. Earthworm activity: A source of potential disturbance of archaeological sediments. *Am. Antiq.* **48,** 277–289 (1983).
17. Canti, M. G. Earthworm activity and archaeological stratigraphy: a review of products and processes. *J. Archaeol. Sci.* **30,** 135–148 (2003).
18. Pessenda, L., Gouveira, S. & Aravena, R. Radiocarbon dating of total soil organic matter and humin fraction and its composition with 14C ages of fossil charcoal. *Radiocarbon* **43**, 595-601 (2001).
19. Nesbitt, H. W. & Young, G. M. Early Proterozoic climates and plate motions inferred from major element chemistry of lutites. *Nature* **299**, 715-717 (1982).
20. Prümers, H., Jaimes Betancourt, C. & Plaza Martinez, R. Algunas tumbas prehispanicas de Bella Vista, Prov. Iténez, Bolivia. *Zeitschrift für Archäologie Außereuropäischer Kulturen*, **1**, 251-284 (2006).
21. Prümers, H. Sitios prehispánicos con zanjas en Bella Vista, Provincia Iténez, Bolivia in *Amazonía: Memorias de las Conferencias Magistrales del 3er Encuentro Internacional de Arqueología Amazónica* (ed. Rostain, S.) 73-89 (IFEA, FLASCO, MCCTH, SENESCYT, 2014).
22. Rodrigues, L., Lombardo, U., Fehr, S., Preusser, F. & Veit, H. Pre-Columbian agriculture in the Bolivian Lowlands: Construction history and management of raised fields in Bermeo. *Catena*, **132**, 126-138 (2015).
23. Walker, J. H. Agricultural change in the Bolivian Amazon (University of Pittsburgh, 2004).
24. Rodrigues, L., Lombardo, U., Trauerstein, M., Huber, P., Mohr, S. & Veit, H. An insight into pre-Columbian raised fields: the case of San Borja, Bolivian lowlands. *Soil*, **2**, 367-389 (2016).
25. Rodrigues, L., Lombardo, U. & Veit, H. Design of pre-Columbian raised fields in the Llanos de Moxos, Bolivian Amazon: Differential adaptations to the local environment? *Journal of Archaeological Science: Reports*, **17**, 366-378 (2018).
26. Dougherty, B. & Calandra, H. A. Prehispanic human settlement in the Llanos de Moxos, Bolivia. *Quaternary of South America and Antarctic Peninsula*, **2**, 163-199 (1984).
27. Prümers, H. & Jaimes Betancourt, C. 100 años de investigación arqueológica en los Llanos de Mojos. *Arqueoantropológicas*, **4** 11-54 (2014).
28. Jaimes Betancourt, C. La cerámica de la Loma Salvatierra (PhD thesis, Universität zu Bonn, 2010).
29. Walker, J. H. Raised field abandonment in the Upper Amazon. Culture & *Agriculture*, **22**, 27-31 (2000).
30. Lombardo, U., Szabo, K., Capriles, J. M., May, J. H., Amelung, W., Hutterer, R., Lehndorff, E., Plotzki, A. & Veit, H. Early and middle Holocene hunter-gatherer occupations in western Amazonia: the hidden shell middens. *PLoS One*, **8**, e72746 (2013).
